# Computational Characterization of the Interaction of CARD Domains in the Apoptosome

**DOI:** 10.1021/acs.biochem.4c00583

**Published:** 2025-01-06

**Authors:** Rita Ortega-Vallbona, Linda Johansson, Laureano E. Carpio, Eva Serrano-Candelas, Sayyed Jalil Mahdizadeh, Howard Fearnhead, Rafael Gozalbes, Leif A. Eriksson

**Affiliations:** † ProtoQSAR SL, Parque Tecnológico de Valencia, Paterna, Valencia 46980, Spain; ‡ Department of Chemistry and Molecular Biology, University of Gothenburg, Göteborg 405 30, Sweden; § Moldrug AI Systems SL, Olimpia Arozena Torres 45, Valencia 46018, Spain; ∥ Pharmacology and Therapeutics, National University of Ireland Galway, Galway H91 TK33, Ireland

## Abstract

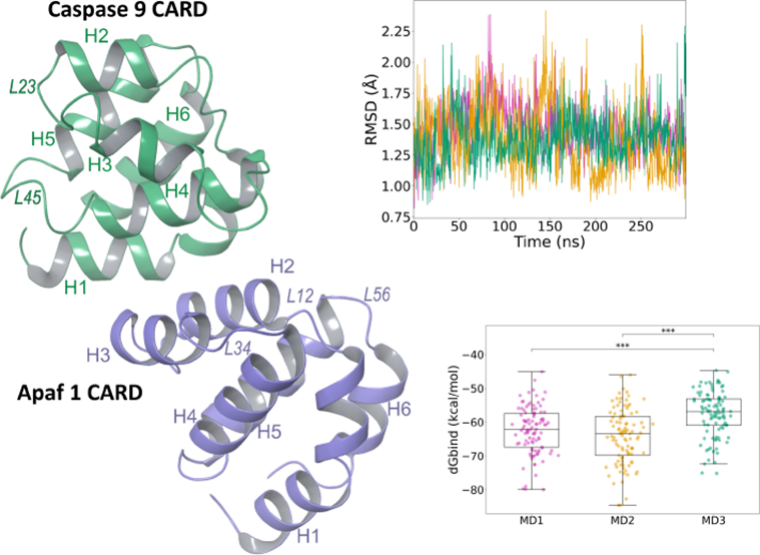

The apoptosome, a critical protein complex in apoptosis regulation, relies on intricate interactions between its components, particularly the proteins containing the Caspase Activation and Recruitment Domain (CARD). This work presents a thorough computational analysis of the stability and specificity of CARD–CARD interactions within the apoptosome. Departing from available crystal structures, we identify important residues for the interaction between the CARD domains of Apaf-1 and Caspase-9. Our results underscore the essential role of these residues in apoptosome activity, offering prospects for targeted intervention strategies. Available experimental complex structures were able to validate the protein–protein docking consensus approach used herein. We furthermore extended our analysis to explore the specificity of CARD–CARD interactions by cross-docking experiments between apoptosome and PIDDosome components, between which there should not be any interaction despite belonging to the same death fold subfamily. Our findings indicate that native interactions within individual complexes exhibit greater stability than the cross-docked complexes, emphasizing the specificity required for effective protein complex formation. This study enhances our understanding of apoptotic regulation and demonstrates the utility of computational approaches in elucidating intricate protein–protein interactions.

## 1 Introduction

Apoptosis is a process of controlled cell death that involves the deliberate elimination of specific cells for the benefit of the organism. It plays a crucial role in embryonic development, organ formation, and homeostasis.^1,2^ Apoptosis also has a significant role in pathological conditions like neurodegenerative diseases, cancer, autoimmune diseases or drug-induced toxicity.^2−5^

There are two pathways by which apoptosis occurs: the extrinsic pathway, initiated by death receptors, and the intrinsic pathway, which involves the mitochondria.^3^ Both pathways have two phases: activation and execution. In the activation phase, a death signal triggers the activation of initiator caspases, that in turn activate the effector caspases. In the execution phase, the effector caspases act on specific cellular structures to induce cell death.^1,6^

During the activation phase of the intrinsic pathway, the most significant event is the apoptosome formation. The apoptosome is a complex of multiple proteins that acts as the platform to activate the initiator Caspase-9^5^. The essential protein of the apoptosome is the apoptotic protease-activating factor (Apaf-1), which has a caspase recruitment domain (CARD) located at the N-terminus connected to a nucleotide-binding domain, followed by two WD40 domains (also known as beta-transducin repeat) located at the C-terminal.^7^

The activation of Apaf-1 occurs when cytochrome c is released from the mitochondria, triggered by stress signals. The binding of cytochrome c to Apaf-1 requires a structural reorganization of the WD40 domains, which in turn enables hydrolysis of the bound nucleotide and the oligomerization of seven Apaf-1 molecules into a wheel-like structure.^5,7^ During this process, the CARD domain of each monomer, which is attached to the nucleotide-binding domain through a long linker segment, no longer becomes restrained and is thereby able to interact with CARDs of other Apaf-1 monomers of the apoptosome and the CARDs of procaspase-9. The CARD–CARD interactions activate Caspase-9, which subsequently activates Caspase-3 and leads to the execution phase of apoptosis.^7−9^

Several experimental and structural studies have investigated CARD–CARD interactions within the apoptosome. The structure of the Apaf-1 and Caspase-9 CARDs has been resolved, revealing that they form a multimeric disk-like assembly within the apoptosome. This configuration is essential for Caspase-9 activation, as it provides a stable platform for procaspase binding.^9−11^ Structural studies using techniques such as X-ray crystallography and cryo-electron microscopy have clarified the specific electrostatic and hydrophobic interactions that stabilize these CARD–CARD assemblies.^10,12−14^

Computational approaches have provided complementary insights into the structural and functional roles of CARD–CARD interactions within the apoptosome. Structural modeling studies revealed that seven Apaf-1 CARDs and four Caspase-9 CARDs form an asymmetric ring, which is essential for stabilizing Caspase-9 and facilitating its activation within the apoptosome core.^14−16^ However, while some of these studies utilized molecular dynamics to refine docked configurations, they often lacked a comprehensive analysis of the dynamic behavior of the complex, leaving the specific contributions of individual interdomain interactions underexplored.

Antiapoptotic compounds have gained a lot of interest in preventing apoptosis in various diseases and in connection with drug-induced toxicity.^4,17−23^ Similarly, the concept of inducing tissue-specific cell death is also becoming more prominent.^24,25^ In order to comprehend the mechanism by which the apoptosome triggers the activation of Caspase-9, it is crucial to investigate the CARD–CARD interactions within this multiprotein complex. This understanding could also pave the way for developing therapeutic interventions for conditions resulting from dysregulation of apoptosis.

To examine the specificity of CARD–CARD interactions within apoptotic complexes, we performed extensive protein–protein docking studies using CARD-containing proteins from both the apoptosome and the PIDDosome, a separate multiprotein complex that also activates caspases in response to cellular stress. The PIDDosome, known for initiating Caspase-2 activation upon DNA damage, relies on CARD interactions in a manner similar to the apoptosome, but within a distinct signaling pathway.^26,27^ This comparative approach, which, to our knowledge, has not yet been undertaken computationally or experimentally, offers insights into whether CARD domains exhibit strict binding preferences within their respective complexes or display broader binding capabilities.

The current study presents a comprehensive analysis of the CARD–CARD interactions that occur in the apoptosome. An integrated computational approach was utilized to evaluate the stability of this interaction and identify the important residues involved. To further explore the specificity of this interaction, protein–protein docking consensus approach was used to evaluate the likelihood of interactions between the apoptosome CARD domains and those of the PIDDosome. Our findings showcase how computational approaches can predict and characterize protein–protein interactions to provide further insights into the selectivity within the CARD death fold subfamily.

## 2 Material and Methods

### 2.1 Data Retrieval

To identify experimentally obtained protein structures containing the CARD domains of Apaf-1 (ApCARD) and Caspase-9 (C9CARD), a search on Uniprot (https://www.uniprot.org/)^28^ for Apaf-1 and Caspase-9 was conducted to identify PDB entries containing both proteins. The resulting six crystal structures with PDB IDs 3YGS,^12,29^ 3J2T,^30,31^ 4RHW,^10,32^ 5JUY,^33,34^ 5WVC^13,35^ and 5WVE^16,36^ were retrieved from the Protein Data Bank (www.rcsb.com).^37^

The crystal structures of the CARD domains of RAIDD (RdCARD) and Caspase 2 (C2CARD) in the PIDDosome were also retrieved from the Protein Data Bank, with PDB IDs 3CRD^38,39^ and 1PYO,^40,41^ respectively.

### 2.2 Protein Structure Analysis

The CARD domain sequences were aligned using MAFFT version 7^42^ with the G-INS-1 method, which performs global alignment based on the Needleman-Wunsch algorithm, requiring truncation of flanking regions to focus on the target domain. Sequence identity was calculated by BioEdit (version 7.7.1).^43^ Aligned sequences were colored in Jalview^44^ by conservation—highlighting conserved physicochemical properties based on intensity^45^—and with the Zappo scheme, which colors residues by physicochemical properties.

The buried solvent-accessible surface area (SASA) in apoptosome CARD domain complexes was computed in UCSF ChimeraX version 1.7^46^ using the formula:
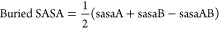
1where sasaA and sasaB are the SASA of each domain, and sasaAB is the SASA of both domains together in the complex.

Electrostatic potential maps were visualized by UCSF ChimeraX’s surface tool using standard parameters. Surface coloring reflects Coulombic electrostatic potential, calculated from atomic partial charges and coordinates according to Coulomb’s law:
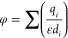
2where φ is the potential, *q_i_* are atomic partial charges, *d_i_* are atomic distances, and ε is the dielectric constant. Potential is given in kcal/(mol·e) at 298 K.

### 2.3 Protein Preparation

The Protein Preparation Workflow tool^47^ in Maestro Schrödinger version 13.7.125 was used to prepare the crystallized structures. This tool automatically adds missing hydrogen atoms and corrects metal ionization states and bond orders to HET groups. It also adjusts the protonation states of ionizable residues, fixes transpositions of heavy atoms, and refines hydrogen bond networks using restrained minimization. Any missing side chains or loops were also filled automatically, and for atoms with multiple occupancies, the position maintaining the highest occupancy was selected.^48^

### 2.4 Molecular Dynamics (MD) Simulations

MD simulations were performed using the Desmond MD simulator engine^49^ implemented in Schrödinger Maestro. All simulations were executed with the same settings and performed in triplicate. First, the System Builder tool was used to prepare the complexes for simulation. The TIP3P force field was adopted to model water molecules,^50^ and the boundary cubic box was set to at least 10 Å spacing for each atom in the protein complex. The simulation box was neutralized by adding proper counterions (Cl-/Na+), and salt concentration was set to 150 mM to simulate physiological conditions. The simulations were conducted under the NPT ensemble, with temperature (300 K) controlled using the Nose-Hoover thermostat^51^ with a relaxation time of 1 ps, and pressure (1 atm) maintained using the Martyna-Tobias-Klein barostat^52^ with a relaxation time of 2 ps and isotropic coupling style.

OPLS4 force filed^53^ was chosen as force field for the protein complex simulations based on its enhanced parametrization of nonbonded interactions. OPLS4 has shown an ability to reproduce experimental data, such as binding free energies and interaction potentials, making it well-suited for predicting protein–protein interactions.^54−56^ The simulation time was set to 300 ns with a time step of 2 fs. A different seed was used in each of the triplicate MD simulations.

### 2.5 Trajectory Analysis and Calculations

The trajectories generated by the MD simulations were analyzed and interpreted using various analytical tools provided by Maestro. Root Mean Squared Deviation (RMSD) was utilized to determine the stability of the simulated protein complexes. The reference position for the calculation of the RMSD was the first frame of the simulation.

Furthermore, postsimulation analysis, including Molecular Mechanics Generalized Born Surface Area (MMGBSA) calculations for the free energy of binding estimation, were conducted.^57^ We computed MMGBSA energy values at every 3 ns of the simulation of each trajectory.

The Simulation Interaction tool in Maestro was used to identify binding residues and analyze their interactions on the most representative structure from each MD simulation. In addition, a Python script was employed to quantify the frequence of occurrence and stability of these interactions throughout the MD simulations. The percentage of native interactions over time in the MD simulations of the complexes was calculated using the crystallized ApCARD-C9CARD structure as a reference 4RHW. For the ApCARD-C9CARD complex simulations, this percentage reflects residue pairs specifically involved in the native interaction. In the cross-docked complexes, however, the calculation of native interaction percentages only considers residues corresponding to the CARD domains from the apoptosome.

For the ApCARD-C9CARD complex, the alanine scanning calculations in Maestro were also conducted to identify key residues contributing the most to the binding affinity.

### 2.6 Protein–Protein Docking

A protein–protein docking consensus approach^56^ was conducted on the benchmarking of the apoptosome and on each cross-docking. Five protein docking engines were used: GRAMM-X,^58^ HDOCK,^59^ HADDOCK,^60,61^ ClusPro,^62,63^ and LZerD.^64^ All dockings were performed using default settings and without templates.

For each docking analysis, the residues relevant to the protein–protein interaction were specified. In the case of the apoptosome CARD domains, the relevant residues were characterized as detailed in the present study. For the PIDDosome CARD domains, the relevant residues were identified based on previous research conducted in our research team. Specifically, the key residues for the PIDDosome interactions were identified to be Glu2, Lys6, Gly34, His40, and Arg64 for RdCARD, and Leu9, His44, Asp58, His61, Cys79, and Asp80 for C2CARD.

The top 10 complexes generated from each docking engine were clustered using the Clustering of Conformers tool in Schrödinger Maestro. The clustering was conducted based on the RMSD of all heavy atoms, hydrogen bonds, and sulfate bonds. Kelley penalty plots were traced to determine the optimal number of clusters.^65^ The complex closest to the centroid of the biggest cluster was then selected for each complex as a representative for further investigation.

### 2.7 Statistical Analysis

The statistical analysis of RMSD curves and MMGBSA values across the triplicate MD simulations of the different CARD domains was performed following a rigorous multistep approach. First, the distribution of each data set was assessed for normality using the Kolmogorov–Smirnov,^66^ Shapiro-Wilk,^67^ and Anderson-Darling^68^ tests, applying a consensus approach where all tests must agree to confirm normality at a significance level of 0.05. For pairwise comparisons of RMSD and MMGBSA values, if both data sets were normally distributed, a Student’s *t* test^69^ was applied; otherwise, the Mann–Whitney U test^70^ was used when one or both data sets deviated from normality, with significance determined at *p* < 0.05.

For the RMSD analysis specifically, to streamline data handling and maintain consistent sampling, we used RMSD values sampled at every 3 ns of each simulation trajectory. This method was also applied to MMGBSA data to achieve comparable representations and analyses across all samples. All statistical analyses were performed using Python (SciPy version 1.11.4)^71^ with both standard and custom functions.

### 2.8 Software Availability

All protein crystal structures were obtained from the Protein Data Bank (https://www.uniprot.org/). The software Schrödinger 2021–1 (Schrodinger LLC, New York, USA; paid license), was utilized for several purposes, including complex clustering, MD simulations, MMGBSA energies, and alanine scanning, following the previously mentioned settings.

For protein–protein docking, five free online platforms were employed, namely HDOCK (http://hdock.phys.hust.edu.cn/), HADDOCK (https://alcazar.science.uu.nl/services/HADDOCK2.2/), ClusPro (https://cluspro.org/home.php), GRAMM-X (https://gramm.compbio.ku.edu/), and LZerD (https://kiharalab.org/proteindocking/), all of which were used with their standard default settings.

## 3 Results and Discussion

### 3.1 Analysis of ApCARD–C9CARD Crystal Structures and Interfaces

**Figure 1:**
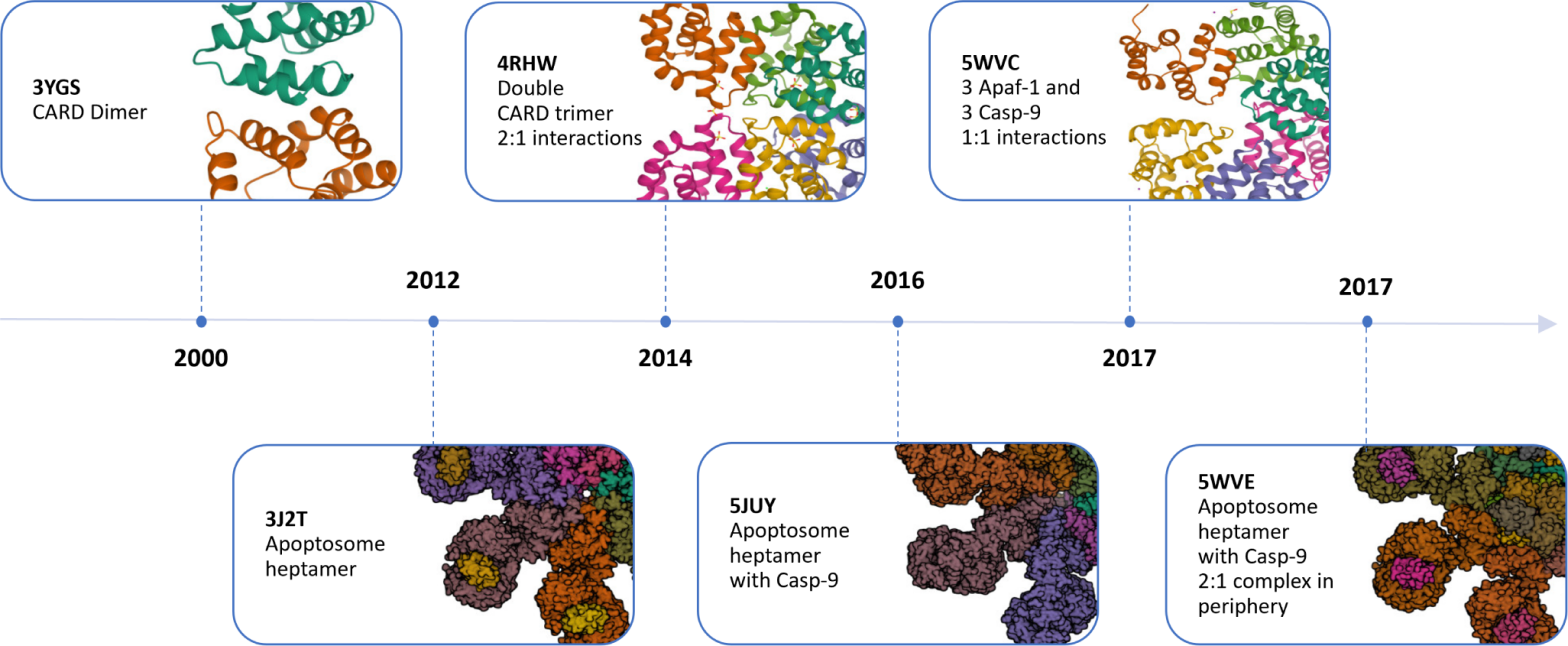
Visual representation of information on six crystal structures of the apoptosome.

We searched the Protein Data Bank for crystal structures that included both ApCARD and C9CARD, either separately or in combination with the entire Apaf-1 protein. It returned six structures, three of which included isolated ApCARD and C9CARD complexes, and three that contained Apaf-1 and other proteins involved in apoptosome formation. Figure 1 and in Table S1 present additional information on each of the six available crystal structures, including their interface types and the interacting residues.

**Figure 2:**
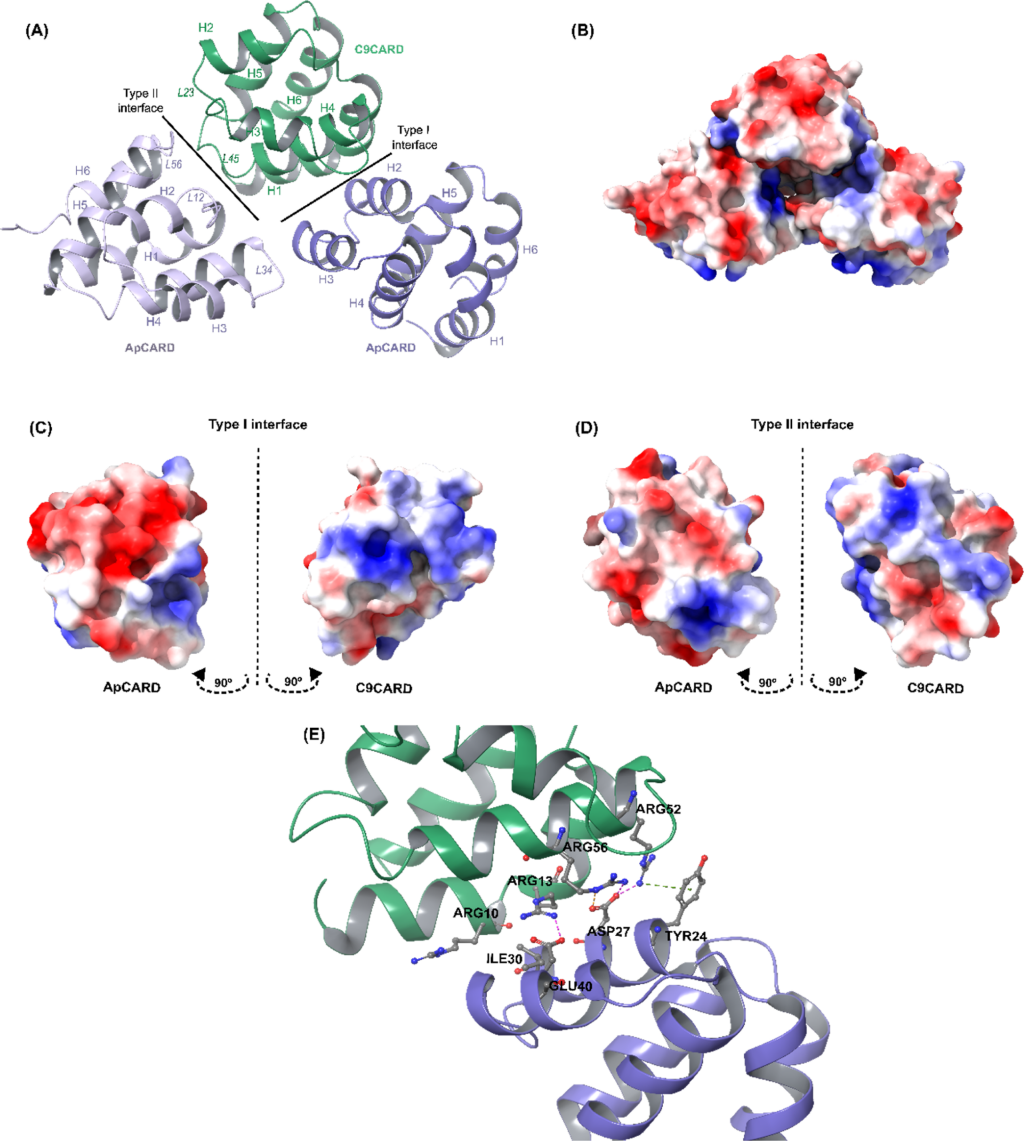
Interaction interfaces between ApCARD (Chains A and B, in light and dark purple, respectively) and C9CARD (Chain E, in green) in the 4RHW complex. (A) The type I and type II interfaces are marked. All α-helices are labeled, with interhelical loops relevant to the type II interface also identified. The loop between helices H1 and H2 is labeled as L12, with other loops following the same naming convention. (B) Chains A, B and E appear represented with their surfaces colored by electrostatic potential. (C) Electrostatic surfaces of ApCARD (chain A, left) and C9CARD (chain E, right) in the type I interface. Domains are rotated 90° to display their interaction surfaces. (D) Electrostatic surfaces of ApCARD (chain B, left) and C9CARD (chain E, right) in the type II interface. Domains are rotated 90° to display their interaction surfaces. (E) Close-up view of the key interacting residues at the type I interface, showing their side chains. Residues are labeled for easier identification within the three-dimensional structure, with key interactions between residues also depicted.

The preliminary investigation of the literature corresponding to the six crystal structures reveals the delineation of two primary interfaces: type I and type II. In the type I interface, with a size of 477.91 Å^2^, C9CARD uses the two α-helices H1/H4 to closely stack against helices H2/H3 from ApCARD, whereas in the type II interface, which has a size of 532.57 Å^2^, C9CARD H4 and interhelical loops L23/L45 contact H6 and loops L12/L56 of ApCARD (Figure 2A). Figure 2B, which shows the combined surface of both domains, illustrates the close interaction between ApCARD and C9CARD. The distribution of red and blue patches across the interface suggests regions of charge complementarity.

When separating ApCARD and C9CARD domains interacting through the type I interface (Figure 2C), the domains appear to exhibit contrasting electrostatic charges at their interaction surfaces. ApCARD shows a predominantly negative charge (red regions), while C9CARD presents a surface with more positively charged (blue) regions. This complementary charge distribution likely facilitates interaction at the type I interface by enhancing electrostatic attraction.

In their type II interaction surfaces shown in Figure 2D, ApCARD again shows regions of negative charge, whereas C9CARD presents a more neutral or less strongly positively charged surface compared to the type I interface. This suggests a possibly weaker electrostatic contribution to binding in the type II interface compared to the type I interface.

The type I interface was selected as the basis for our study, as it is present in all published structures and is the core structure for subsequent interfaces and domain positions. Additionally, it has been reported that the type I interface is likely a characteristic feature of several cell death multiprotein assemblies, including the human PIDDosome.^13^

All the structures presenting the type I interface were carefully examined, and the structure with the best resolution (PDBID: 4RHW, resolution 2.1 Å) was selected for further analysis. This complex is a multimeric assembly, consisting of a double trimer in a 2:1 interaction model of CARD domains. To analyze the interactions in the type I interface, we used chain B (ApCARD) and chain E (C9CARD) from the crystal structure (Figure 2E). All interactions identified between chain B and chain E in the crystallized structure are summarized in Table S2.

### 3.2 ApCARD–C9CARD Interaction Characterization

**Figure 3:**
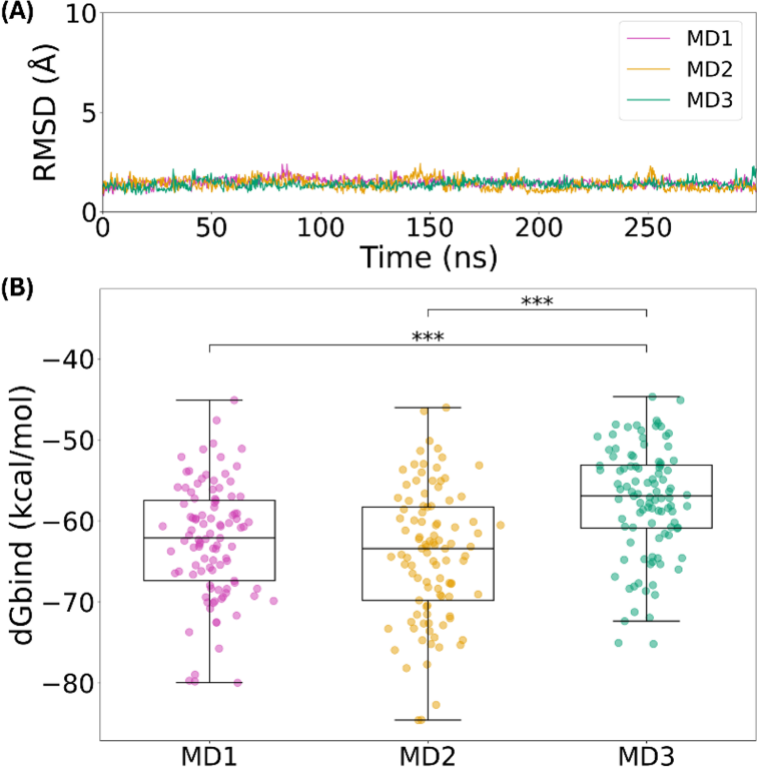
(A) RMSD and (B) MMGBSA calculations from the triplicate MD simulations of the ApCARD–C9CARD type I complex.

Triplicate 300 ns MD simulations of the ApCARD and C9CARD domains interacting through the type I interface were conducted to ensure the stability of the interaction between the two domains. The average RMSD was 1.41 ± 0.22 Å with individual means of 1.46 ± 0.19 Å for MD1, 1.39 ± 0.27 Å for MD2, and 1.38 ± 0.19 Å for MD3. Median RMSD values are shown in Table S3. Figure 3A indicates that the interaction is highly stable, with very similar overall behavior in all three simulations. However, a statistical comparison of the mean RMSD values revealed a significant difference between MD1 and the other two simulations (MD2 and MD3) as shown in Table S4. This analysis indicated that MD1’s average RMSD was significantly higher than those of MD2 and MD3, suggesting slightly greater deviations in stability for MD1 compared to the other simulations.

MMGBSA calculations were performed on the MD trajectories to determine the free energy of the interaction. Figure 3B shows that, in agreement with the overall stability observed in the RMSD plots, the average energy values of the three MD simulations are comparable (−62.44 ± 6.97 kcal/mol for MD1, −64.21 ± 8.07 kcal/mol for MD2, and −57.57 ± 6.74 kcal/mol for MD3). The median values for the MMGBSA are consistent with these averages and are shown in Table S3. While a statistical analysis revealed differences between simulations (see Table S5 for details), the magnitude of these variations was small, with MD3 showing marginally weaker binding on average. These results suggest only minor variations in interaction strength across simulations.

**Figure 4:**
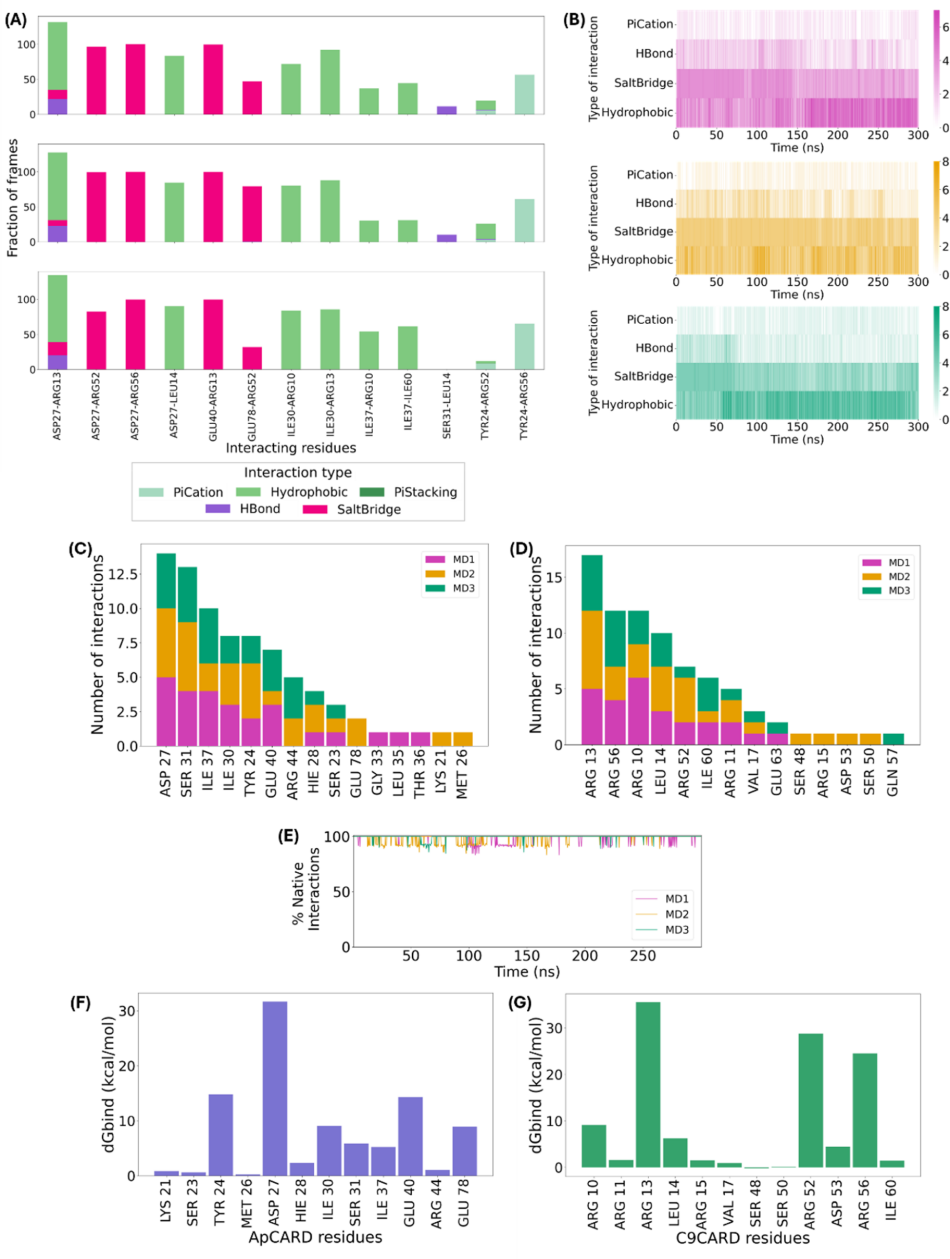
Analysis of most important residues for ApCARD–C9CARD interaction. (A) Percentage of simulation frames in which interacting residues between domains maintain specific interactions. Only interactions present in over 10% of the frames are shown. The top, middle, and bottom plots correspond to MD1, MD2, and MD3, respectively. Values above 100% are possible when residue pairs form multiple simultaneous contacts of different types, such as hydrogen bonds and salt bridges, throughout the simulation; (B) Heatmaps showing the frequency of each interaction type over the 300 ns simulation time: MD1 (magenta), MD2 (yellow), and MD3 (green); (C, D) Number of interactions per residue in the triplicate MDs for (C) ApCARD and (D) C9CARD, respectively; (E) Percentage of native interactions along the simulation time, taking the interactions detected in the crystallized ApCARD-C9CARD type I interface as a reference; (F, G) Alanine scanning results for (F) ApCARD and (G) C9CARD, respectively.

To quantify the interactions between the two domains along the simulation time, the analysis of percentage of frames each interaction is present was conducted (Figure 4A). The most common and stable interactions are hydrophobic and salt bridges, as illustrated by the representation of interaction types in function of simulation time (Figure 4B). The most stable hydrophobic interactions occur between Asp27 of ApCARD and Arg13/Leu14 of C9CARD, and Ile 30/Ile 37 of ApCARD with Arg10, Arg13 and Ile60 of C9CARD, as illustrated in Figure 4A. This stability can be attributed to the complementary physicochemical properties of the interacting residues. The electrostatic attraction between the negatively charged Asp27 and positively charged Arg13 enhances the stability of this contact, creating a strong ionic interaction at the interface. Additionally, the presence of hydrophobic residues, such as Ile30 and Ile37 of ApCARD and Leu14 and Ile60 of C9CARD, promotes clustering through the hydrophobic effect. This clustering minimizes solvent exposure and strengthens the protein–protein interaction. Arginine residues, although primarily charged, contribute to hydrophobic interactions through their aliphatic side chains, which interact favorably with isoleucine residues.

It was also observed that Asp27 of ApCARD forms salt bridges with Arg13, Arg52 and Arg56 of C9CARD. The salt bridge with Arg56 is stable throughout the 300 ns simulation, and around 90% of simulation time with Arg52, while it only occurs with Arg13 in about 10% of the three simulations (Figure 4A). Similarly, Glu40 in ApCARD forms a salt bridge with Arg13, which is stable across all three simulations. Additionally, there is a salt bridge between Glu78 and Arg52, though this interaction is not consistently maintained, disappearing in MD1 and MD3 (Figure S1). The heatmaps in Figure 4B show that the salt bridge interactions behave similarly along the triplicate MD simulations, as they are maintained mostly along the first half of the simulation, and most of them have never been lost.

The stability of these salt bridges can be attributed to the strong electrostatic interactions between the negatively charged residues (Asp27, Glu40, and Glu78) in ApCARD and positively charged arginine residues (Arg13, Arg52, and Arg56) in C9CARD. These oppositely charged pairs are well-positioned at the interface, allowing for a highly favorable ionic attraction. Notably, the rigid structure of the arginine side chains, which contain multiple nitrogen atoms capable of stabilizing the charge, helps to maintain these interactions even under dynamic conditions.

In the observed interaction between Asp27 and Arg13, the hydrophobic nature of their interaction can be explained by the environmental context within the buried region of the interface. In some specific environments, the polar groups of Asp and Arg lose access to water, reducing the ionic interactions typically favored in aqueous conditions. This solvent exclusion effect likely drives the residues to interact via their hydrophobic regions—specifically, the CH_2_ groups in Asp’s side chain and the aliphatic backbone of Arg.^72^ Although Arg is conventionally classified as a polar, charged residue, its side chain includes a nonpolar aliphatic segment that can form hydrophobic-like contacts in certain contexts. Moreover, the structural flexibility and unique guanidinium group of Arg can contribute to nonpolar interactions in environments where hydrogen bonding or ionic pairing is less favorable, as suggested by prior studies on the behavior of Arg.^73^ However, it should also be considered that the observation of hydrophobic interaction between these two polar residues could result from an artifact in the algorithm used by Maestro to estimate interactions rather than reflecting a true physicochemical feature of the interface.

Hydrogen bonds, while less numerous than salt bridges, also contribute to the ApCARD-C9CARD interaction across the three MD simulations. As shown in Figure 4A, Asp27 in ApCARD forms a transient hydrogen bond with Arg13 in C9CARD, complementing their other stable interactions, although this hydrogen bond persists for only about 20% of the simulation time. Additionally, Ser31 in ApCARD forms a brief hydrogen bond with Leu14 in C9CARD during MD1 and MD2, and Tyr24 in ApCARD occasionally forms a hydrogen bond with Arg52 in C9CARD.

The limited stability of these hydrogen bonds compared to salt bridges is likely due to their dependence on precise geometric alignment, which is more susceptible to fluctuations in the molecular dynamic environment. Nevertheless, transient hydrogen bonds can contribute to stabilizing the interface momentarily, particularly when combined with other interactions like salt bridges and hydrophobic contacts.

Finally, Tyr24 of ApCARD engages in π-cation interactions with Arg52/Arg56 of C9CARD. The interaction with Arg56 is particularly stable, persisting for around half of the simulation time with intermittent occurrences, as shown in Figure S1. π-cation interactions between the aromatic ring of Tyr24 and the positively charged side chains of arginine residues contribute to stabilizing the CARD–CARD interface. This interaction is particularly robust due to the complementary electrostatic forces and stacking arrangement, which create an additional stabilizing effect in conjunction with the surrounding hydrogen bonds and salt bridges.

The simulation trajectory clustering was performed to determine the most likely conformation of the two protein domains in each MD simulation. The clustering allowed us to group the most similar conformations and locate the center of the most populated cluster, which represents the most probable conformation of the two protein domains for each MD simulation. In MD1, MD2, and MD3, the most populated clusters contained 37.82%, 38.10%, and 37.90% of the total structures, respectively. Notably, the distributions of structures across the three main clusters were fairly balanced, with the second and third clusters in each trajectory containing 31.93% and 30.25% in MD1, 32.14% and 29.76% in MD2, and 33.06% and 29.03% in MD3. This balanced distribution, along with the low variance in RMSD values, suggests that the conformations sampled during the trajectories do not exhibit significant variation.

Further analysis was conducted on the three most probable structures by superposing them and calculating the RMSD values. The RMSDs between the dominant clusters across the trajectories were 1.57 Å for MD1 and MD2, 1.10 Å for MD1 and MD3 and 2.00 Å for MD2 and MD3. These low RMSD values confirm that the main structures obtained from the triplicate MD simulations are highly similar, which is consistent with the observed stability throughout the simulation period.

To quantify the interactions in the clustered structures, we counted the number of interactions formed by each residue with the opposing protein’s CARD domain. The accumulated interactions for each residue for ApCARD and C9CARD are shown in Figure 4C,D, respectively. By summing the interactions across all three simulations and comparing them with those observed in the crystallized structure, we found that 90% of the interactions in the crystal structure coincide with those observed in the MD simulations, which aligns with the high percentages of native interactions calculated along the MD simulations represented in Figure 4E. For ApCARD, these are Tyr24, Asp27, Ile30, and Ser31, located in the α-helix H2, as well as Ile37 and Glu40, located in H3. In C9CARD, the key residues are Arg10, Arg13, and Leu14 in H1, and Arg52, Arg56, and Ile60 in H4. These residues exhibited more than five accumulated interactions across all three simulations and agree with the interactions seen in the crystallized structure, indicating their consistent involvement in the CARD–CARD interaction (Table S6). However, interactions observed in clustered structures only partially match those from the full MD simulations. Specifically, Asp27, Glu40, Glu78, and Tyr24 of ApCARD interact with Arg13, Arg52, Arg56, and Asp53 of C9CARD predominantly through hydrogen bonds in the clusters, whereas the MD simulations reveal a tendency toward salt bridges or hydrophobic interactions.

The differences arise because MD simulations capture dynamic, fluctuating interactions over time, which are not fully represented in static, clustered structures. These structures, derived by averaging, may emphasize certain stable but less frequent interactions while downplaying transient yet recurrent interactions, thus creating a partial overlap with the MD-detected interactions. Nonetheless, the interaction counts per residue for each domain, as shown in Figure 4C,D, provide a summary of each residue’s relative importance in mediating CARD domain binding.

Upon separate analysis of the three trajectories, the most probable conformation from the most stable molecular dynamics simulation (MD2) was chosen as the basis for further analysis. Using this conformation, we conducted alanine scanning to determine the critical residues for interaction stability between the two domains. Figure 4F,G depict how mutation to alanine of each residue participating in the CARD–CARD interaction impacts the binding free energies. In these figures, we observe that the residues that contribute the most to energy change in ApCARD are (in descending order) Asp27, Tyr24, Glu40, Ile30 and Glu78, and in C9CARD, they are Arg13, Arg52, Arg56 and Arg10.

Although the interaction of ApCARD Arg44 appears significant in the interaction counts (Figure 4C) and in the crystal structure, a closer examination using alanine scanning and the stability of these interactions over simulation time reveals that the interactions established by this residue may not be as relevant as initially suggested.

Based on the data in Figure 4 and the specific interactions detailed in Table S6, we note that the results from interaction counts and alanine scanning align closely. Combining both methods, we conclude that the most important residues for the CARD–CARD interaction in ApCARD are Asp27, Tyr24, Glu40, and Ile30, and in C9CARD, they are Arg13, Arg52, Arg56, and Arg10.

When comparing our findings with the literature, we noticed that in the publication corresponding to the structure with PDB ID 3YGS,^12^ two mutations on the Apaf-1 CARD domain (Asp27Ala on helix H2 and Glu40Ala on helix H3) were identified to eliminate interaction with Caspase-9. The same work also reports that the mutation Ser31Ala weakens but does not eliminate interaction. Our results support these observations, as they show that despite Ser31 being the second most interacting residue, substituting it with alanine results in only a modest reduction in the binding energy of approximately 5 kcal/mol. Moreover, when examining the fraction of simulation time during which Ser31 maintains interactions with C9CARD residues, we found that it does not form stable interactions. Thus, our computational results are in strong agreement with the experimental findings.

Qin et al.^12^ also analyzed which mutations on C9CARD that affect the interaction between the two domains and identified two mutations (Arg13Ala at the hinge region between H1a and H1b helices and Arg56Ala on helix H4) that prevent interaction with wild-type Apaf-1 CARD. Our analyses also emphasize the significance of these two residues. Two additional mutations (Arg11Ala at the end of helix H1 and Arg52Ala on helix H4) were also identified to significantly reduce the interaction.^12^ Our computational characterization confirms the importance of Arg52 for the interaction. However, although Arg11 was involved in five interactions overall, the difference in binding energy caused by its mutation was not significant. This aligns with the absence of Arg11 in Figure 4A, implying that none of its interactions persist for more than 10% of the simulation time. Consequently, we do not classify Arg11 as critical for the interaction based on our computational analysis.

For the structure with PDB ID 4RHW, Hu et al.^10^ and later also Cheng et al.^34^ for crystal structure 5JUY, reported a series of crucial interdomain hydrogen bonds in the type I interface that match the findings of the previously analyzed publication. These bonds were formed between Glu40 of ApCARD and Arg13 of C9CARD, and between Asp27 of ApCARD and Arg52 of C9CARD. Our computational analysis also confirms the significance of these residue pairs for the ApCARD–C9CARD interaction, being present for an average of 99.77% and 92.85% of the simulation time, respectively. However, they are primarily detected as salt bridges rather than hydrogen bonds, suggesting that the atomic distances between these residues were longer during most of the simulation.

### 3.3 ApCARD–C9CARD Consensus Docking

Next, we used the current system to further benchmark our protein–protein docking consensus approach^56^ with the purpose of determining if this (a) could reproduce the experimental pose of this CARD–CARD interaction, and thus (b) could be used to generate reliable complexes also for other interaction pairs in the CARD death fold subfamily.

The same chains from the PDB ID 4RHW that were selected for the previous analysis were also the starting point for the ApCARD–C9CARD docking using the five tools mentioned in the Methods section. This process generated 43 complex poses. By clustering the identified complexes and analyzing the Kelley penalty plot (Figure S2A), we determined that the optimal number of clusters was 5. From this clustering, we obtained two majority clusters: cluster 1 with 15 complexes and cluster 3 with 13 complexes.

**Figure 5:**
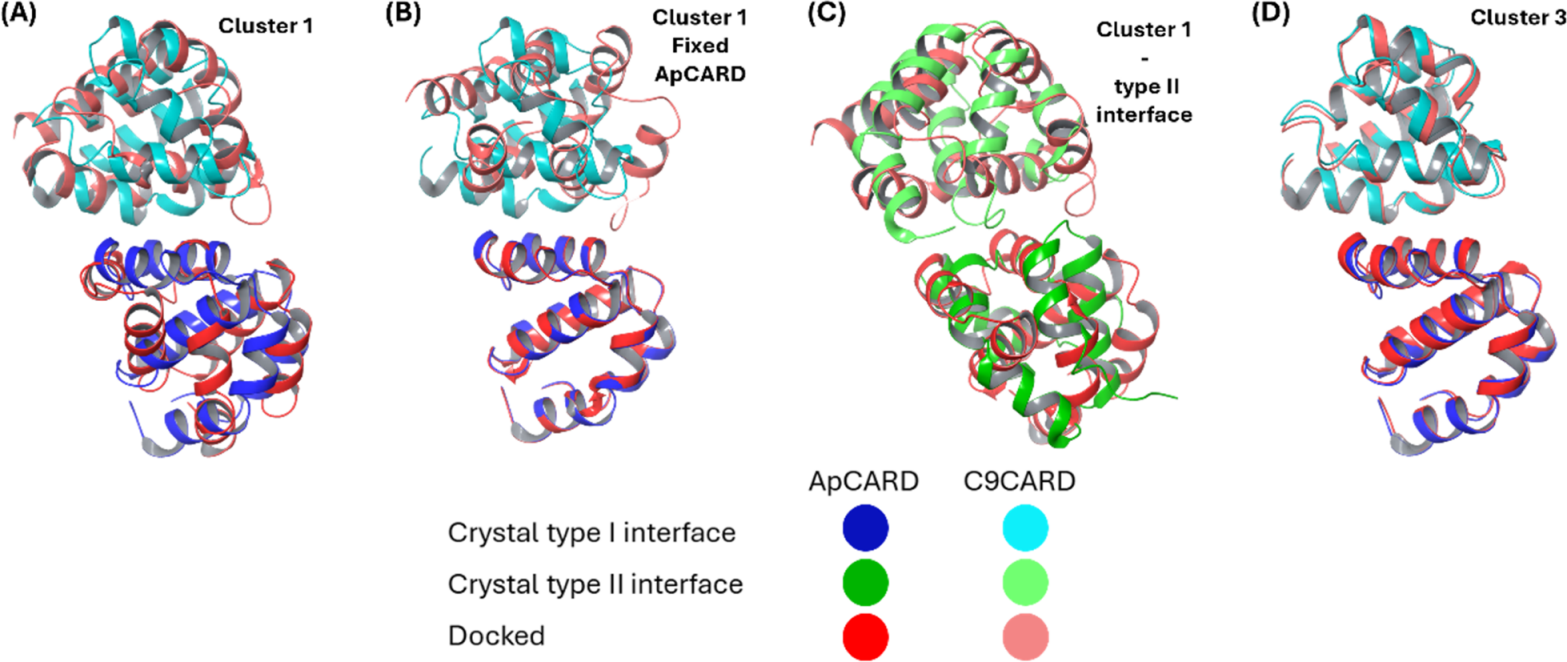
Meta-docking of ApCARD-C9CARD (A) Superposition of the crystallized ApCARD-C9CARD complex (type I interface shown in blue shades) with the docked complex from Cluster 1 (red shades) (B) Superposition of Cluster 1 with the crystallized complex, with ApCARD aligned to highlight the rotation of C9CARD in the docked complex (C) Superposition of the crystallized ApCARD-C9CARD complex (type II interface shown in green shades) with the docked complex from Cluster 1 (red shades) (D) Superposition of the crystallized ApCARD-C9CARD complex (type I interface; blue shades) with the docked complex from Cluster 3 (red shades). In all cases, darker shades correspond to ApCARD; lighter shades correspond to C9CARD.

The complexes closest to the centroid of each of the two clusters were selected and superimposed on the crystallized ApCARD – C9CARD complex. The resulting RMSD for the superimposed structure from Cluster 1 was calculated to be 7.38 Å (Figure 5A). To help in visualizing the difference between the crystallized strucure and that from Cluster 1, the two structures were superposed with ApCARD fixed in place (Figure 5B). In this comparison, C9CARD appears rotated relative to its position in the crystallized complex. Analysis of the Cluster 1 structure (Table S6) shows that 25% of pairwise interactions match those from the crystallized complex. Specifically, ApCARD residues Arg44, Glu40, Ile37, Ile30, and Asp27 interact with C9CARD residues Ile60, Arg13, Arg10, and Arg56. Given this matching of interactions, it is probable that an MD simulation of Cluster 1 could stabilize it into a pose closer to the crystallized structure.

We compared the Cluster 1 pose with the crystallized ApCARD–C9CARD complex interacting through the type II interface (Figure 5C) to determine if Cluster 1 placed C9CARD closer to its type II position relative to ApCARD. The resulting RMSD of 15.29 Å confirms that the Cluster 1 pose does not correspond to the type II interface, which was expected since the docking process specifically targeted residues relevant to the type I interface, although no templates were used for any of the dockings.

On the other hand, the superimposition of the Cluster 3 structure with the type I interface crystallized structure resulted in an RMSD of 0.65 Å (Figure 5D). This result, with an RMSD below the threshold of 2 Å, shows that the meta-docking approach can reach poses very close to what is obtained experimentally.

### 3.4 RdCARD–C9CARD Cross-Docking Characterization

Having confidence that the meta-docking approach was able to generate reliable CARD–CARD complexes, we then performed cross-dockings to evaluate the specificity of interaction between CARD domains from different protein complexes. To this end, we docked the RAIDD CARD domain (RdCARD) from the PIDDosome (PDB ID 3CRD) with C9CARD from the apoptosome. The RAIDD CARD domain interacts with the CARD domain of Caspase-2 (C2CARD), which is unknown whether it could also interact with the CARD domain of Caspase-9. A total of 50 complexes were generated, ten from each docking engine, which were clustered into 11 clusters, as indicated by the Kelley penalty plot (Figure S2B). Cluster 2, containing 19 poses, was the largest, and the closest structure to the centroid therein was selected for subsequent calculations.

**Figure 6:**
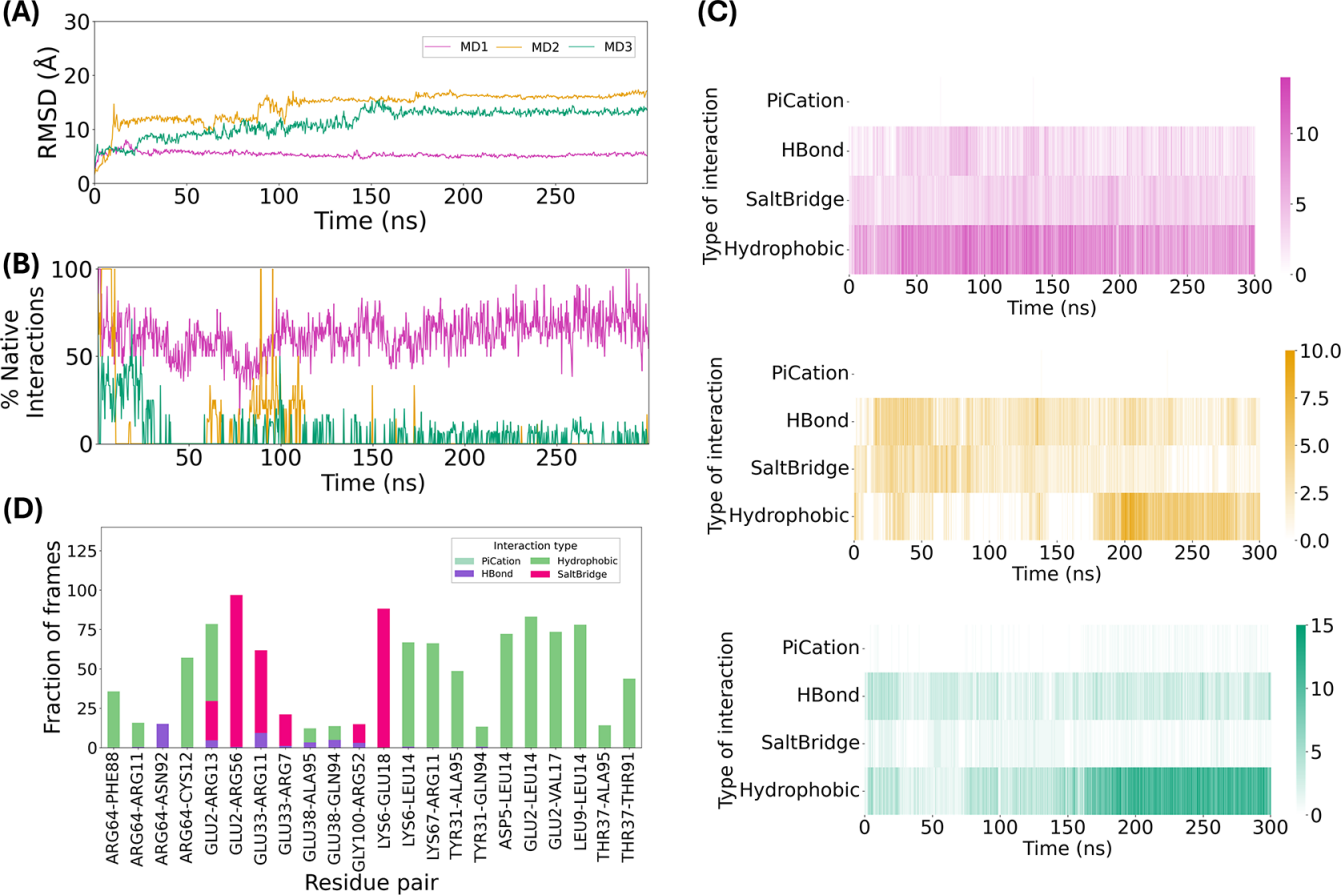
Consensus docking results for RdCARD-C9CARD. (A) RMSD calculation of the complex along the triplicate MD simulations; (B) Percentage of native interactions over time taking residues of C9CARD as a reference; (C) Heatmaps showing the frequency of each interaction type over the 300 ns simulation time: MD1 (magenta), MD2 (yellow), and MD3 (green); (D) Interaction fraction of each interacting pair of residues in MD1.

We performed 300 ns MD simulations in triplicate to assess the stability and interactions in this complex. The average RMSD of the three simulations was 10.34 ± 4.28 Å, with MD2 and MD3 clearly shifting positions as the simulations progressed, Figure 6A. The representation of the percentage of native interactions (Figure 6B) and the heatmaps showing the number of interactions by type along the 300 ns of simulation (Figure 6C) all support this observation, as MD2 and MD3 change their pattern of interactions as the time of simulation progresses. Detailed heatmaps showing the change of interaction pattern for MD1, and for MD2 and MD3 are shown in Figures S3 and S4, respectively.

Only one of the three simulations (MD1) indicated a stable complex, with an average RMSD of 5.38 ± 0.47 Å, which is yet significantly higher than the average RMSD observed across all three MD simulations of the native ApCARD-C9CARD complex, as shown in Table S4. Table S3 shows that MD1 had a median RMSD of 5.31 Å, closely matching its mean, while MD2 and MD3 had medians of 15.51 and 12.57 Å, respectively, consistent with their higher mean values, although the distributions are not Gaussian.

The free energy of interaction in the one stable system (MD1) is −62.08 ± 11.85 kcal/mol, which, interestingly, is not significantly different from the average free energies of MD1 and MD2 for the native ApCARD-C9CARD complex, as shown in Table S5.

Upon further investigation of the final structure from MD1, we observed that the pose changes early in the simulation, causing a 5 Å RMSD deviation almost immediately, stabilizing thereafter. In Figure S5, which shows the superposition of the first and last frames of this simulation fixing the C9CARD chain, we show that RdCARD shifts toward helix H6, and interacts with both helix H1 and helix H6, indicating a reorientation of the interface. This new interface does not match with the canonical Type I and Type II interfaces observed in the native ApCARD-C9CARD complex, where C9CARD utilizes helixes H1 and H4 (Type I interface), and helix H4 and loops L45 and L23 (Type II interface), as described above.

Coming back to the percentage of native interactions, we observed that the values along MD1 of the RdCARD-C9CARD complex are lower than those of the native ApCARD-C9CARD interaction, which, even when taking both residues of the pair into account are always higher than 80% for the three MD simulations, and, when considering only the residues of ApCARD or C9CARD as a reference, are above 90% for the majority of the simulation time. For MD1 of the RdCARD-C9CARD complex, the percentage of native interactions is much more variable and remains in the range of 50%–80% for most of the simulation time.

In the MD1 simulation trajectory, residues Arg11, Arg13, Cys12, Glu18, Leu14, Val17, Arg56, Thr91 and Ala95 in C9CARD primarily interact with Glu2, Asp5, Lys6, Leu9, Glu33, Arg64, and Lys67 of RdCARD (Figure 6D). Among these, Arg13, and Arg56 in C9CARD also emerged as critical residues for ApCARD-C9CARD interactions. Notably, Leu14 of C9CARD interacts with four residues of RdCARD for approximately 75% of the simulation time. Although Leu14 was not classified as a key residue in ApCARD-C9CARD interactions, it maintained interactions with Asp27 of ApCARD for an average of 78% of the simulation time across all three MD simulations (Figure 4A). In addition, Leu14 was identified as one of the residues with the highest number of interactions in the most representative structures from the three MD simulations (Figure 4D).

The analysis of the heatmap in Figure S3 indicates that the hydrophobic interactions between several RdCARD residues and C9CARD’s Leu14 are quite stable throughout the simulation, which aligns with the nonpolar affinity of leucine that favors sustained interactions in hydrophobic environments. Similarly, the salt bridge between RdCARD’s Glu2 (negatively charged) and C9CARD’s Arg56 (positively charged) remains stable over time, as the electrostatic attraction between charged residues typically strengthens the stability of such interactions. However, the shift in the interface is evident from new interactions with C9CARD residues in helix H6, including Phe88, Thr91, Asn92, and Ala95, as well as Arg11, Cys12, Val17, and Glu18 in helix H1. These contacts suggest that RdCARD has displaced toward H6, moving away from stable interactions with H4. The transient interactions of RdCARD’s Met1, Glu2, Pro98, and Gly100 with C9CARD’s Arg52 further support this shift, as these residues lack the stability required for sustained binding. Only the salt bridge RdCARD’s Glu2 and C9CARD’s Arg56 (located in H4) remains stable.

Considering the instability observed in the two additional MD simulations of the RdCARD-C9CARD complex, along with the pose shift detected in MD1, we conclude that C9CARD does not interact with RdCARD as it does with ApCARD. This difference aligns with expectations, as these cross-docked domains lack a biological context for interaction.

### 3.5 ApCARD–C2CARD Cross-Docking Characterization

A second cross-docking experiment was performed by combining ApCARD from the apoptosome with the Caspase-2 CARD (C2CARD) from the PIDDosome. This process generated 50 poses, further grouped into 14 clusters according to the Kelley penalty plot (Figure S2C). Out of these 14, two large clusters, Cluster 5 with eight complexes and Cluster 8 with seven complexes, were identified. Since both clusters had a similar number of structures, we selected the complex closest to the centroid of each of the two clusters to proceed with our analysis.

**Figure 7:**
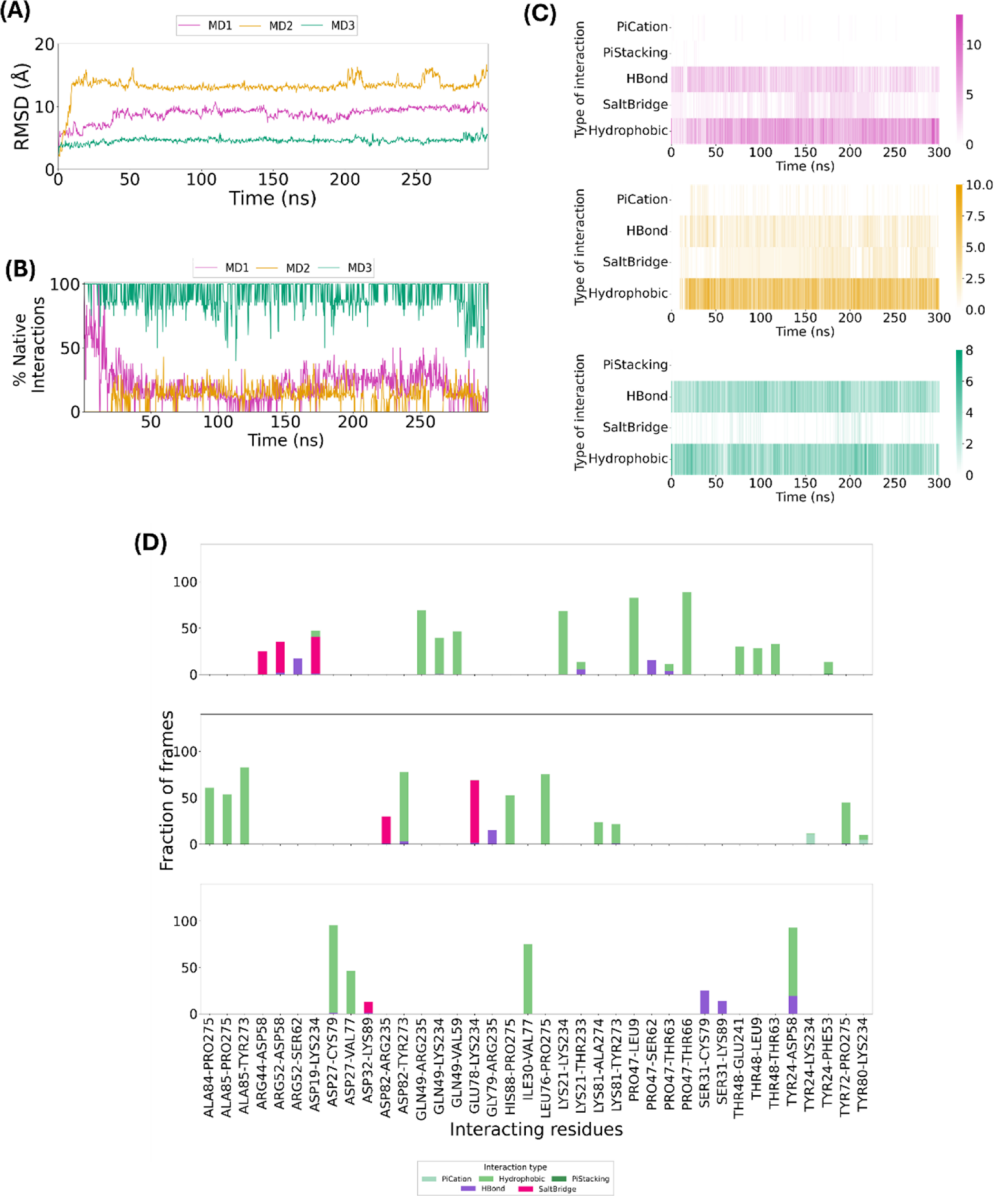
Consensus docking results for the ApCARD-C2CARD Cluster 5 complex. (A) RMSD calculation of the complex along the triplicate MD simulations;(B) Percentage of native interactions over time taking residues of ApCARD as a reference; (C) Heatmaps showing the frequency of each interaction type over the 300 ns simulation time: MD1 (magenta), MD2 (yellow), and MD3 (green); (D) Interaction fraction of the triplicate MD simulations. The top, middle and bottom plots correspond to MD1, MD2 and MD3 respectively.

For the Cluster 5 complex, the average RMSD was 8.88 ± 3.71 Å. Individually, MD1 and MD2 exhibited higher RMSD values, 8.82 ± 1.13 Å and 13.23 ± 1.56 Å respectively, indicating substantial deviation from the initial structure. In contrast, MD3 showed the highest stability of the three MD simulations, with an average RMSD of 4.59 ± 0.40 Å, Figure 7A. Consequently, the MMGBSA energy for the triplicate MD simulations on the Cluster 5 structure varies significantly between the three simulations. MD1 showed an average MMGBSA energy of −48.70 ± 8.70 kcal/mol, while MD2 had −36.04 ± 10.02 kcal/mol, and MD3 presented the least favorable binding energy at −24.76 ± 5.69 kcal/mol. The medians of both RMSD and energy values are consistent with the means and are shown in Table S3.

Notably, MD3 maintained a high percentage of native interactions throughout the entire 300 ns simulation, as depicted in Figure 7B, prompting us to examine the positioning of C2CARD relative to ApCARD at the start and end of the simulation, as shown in Figure S6. We observed that C2CARD shifts toward helix H5, establishing contacts with both helix H2 and helix H5. This interface, similar to that observed in the RdCARD-C9CARD complex, does not align with any of the known native CARD–CARD interfaces within the apoptosome, described in Section 3.1.

The high percentages of native interactions in MD3 can be attributed to the limited number of interacting residue pairs. As depicted in the heatmap in Figure S7C, only six residue pairs interact for most of the simulation, and the ApCARD residues involved in these stable interactions—Asp27, Ile30, Ser3, and Tyr24—are all key stabilizing residues in the native ApCARD-C9CARD complex. Figure 7C shows that the majority of these interactions are hydrogen bonds and hydrophobic contacts, which are generally weaker than salt bridges or cation-π interactions. This explains the less favorable binding energy observed for this pose. In more detail, in the MD3 simulation, Asp27 of ApCARD interacts with Cys79 and Val77 of C2CARD during 95% and 46% of the simulation time, respectively. Additionally, Ile30 of ApCARD maintains hydrophobic interactions with Val77 of C2CARD for 74% of MD3. Hydrogen bonds were also identified between Ser31 of ApCARD and both Cys79 and Lys89 of C2CARD, though these interactions occurred for less than 25% of the MD3 simulation time. Notably, none of these interactions are conserved in the other two MD simulations for Cluster 5. Tyr24 of ApCARD consistently participates in interactions during MD3, engaging for 92% of the simulation time. Tyr24 also emerges as the residue involved in the most interactions across all three simulations. In MD2 of Cluster 5, the only notable interaction involves Glu78 of ApCARD, which forms a salt bridge with Lys234 of C2CARD for 68% of the simulation time. However, MD1 of Cluster 5 lacks significant interactions between C2CARD and the ApCARD residues crucial for native CARD–CARD interactions in the apoptosome.

**Figure 8:**
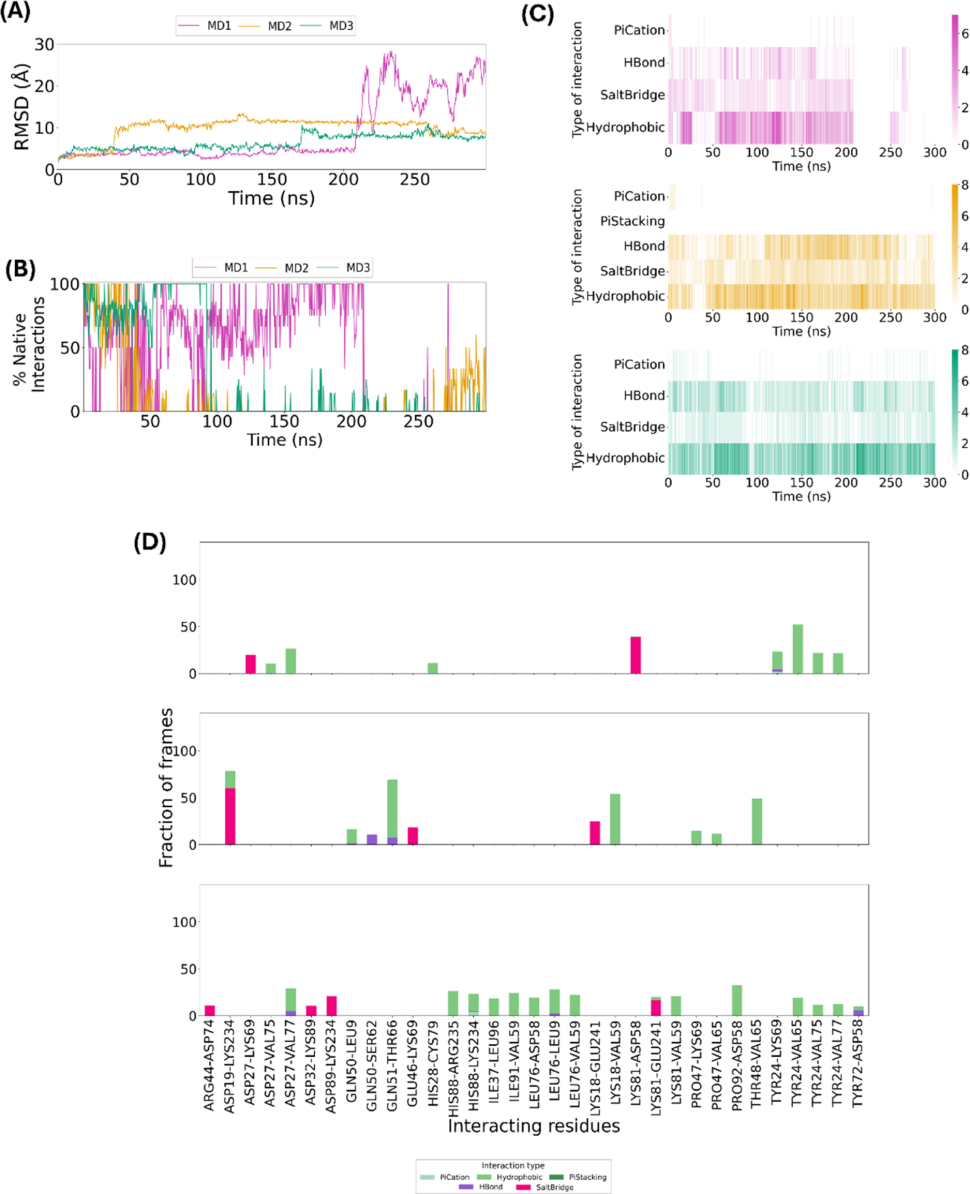
Consensus docking results for the ApCARD-C2CARD Cluster 8 complex. (A) RMSD calculation of the complex along the triplicate MD simulations;(*B*) Percentage of native interactions over time taking residues of ApCARD as a reference; (*C*) Heatmaps showing the frequency of each interaction type over the 300 ns simulation time: MD1 (magenta), MD2 (yellow), and MD3 (green)*; (D)* Interaction fraction of the triplicate MD simulations. The top, middle and bottom plots correspond to MD1, MD2 and MD3 respectively.

When analyzing the interactions of both Cluster 5 and Cluster 8 complexes across their triplicate MD simulations, as depicted in Figure 8, we observed significant variation in the key interacting residue pairs between the three simulations for each complex.

Regarding the complex from Cluster 8, the average RMSD across all MD simulations was 8.25 ± 4.95 Å, with a median of 7.68 Å, indicating a lower overall deviation than that observed for Cluster 5. However, this is only due to MD1 and MD3 being stable (but with a much higher RMSD, 8.60 ± 7.58 Å and 6.35 ± 1.73 Å, respectively, than the native complex) for the first ∼170 ns. MD2 dissociates completely, and the remaining two complexes have RMSDs in the range of 7–12 Å (Figure 8A). Regarding MMGBSA calculations of the binding energy, the average value of −23.17 ± 12.92 kcal/mol is higher than the average for the Cluster 5 complex and also agrees with the poor interactions. Table S3 provides the means and medians of these metrics.

The instability of these three simulations is evident in the substantial fluctuations in the percentage of native interactions over the 300 ns simulations depicted in the Figure 8B, which is also illustrated by the varying patterns in the interaction heatmaps in Figure 8C. Figure S8 provides a detailed view of the interaction patterns between specific residue pairs across the three MD simulations, highlighting the continuous repositioning and rearrangement of residues from the two CARD domains as they interact dynamically with each other.

For the three simulations performed with the Cluster 8 complex, interactions are generally less stable, with few interactions persisting for more than 10% of the simulation time (Figure 8D). Even the most stable interactions in Cluster 8 occur in less than 50% of the simulation time. An exception is MD2, where, after the CARD domains detach from their initial docked positions, they adopt a different pose stabilized by four ApCARD residues, none of which are identified as important in apoptosome CARD–CARD interactions. In MD1 and MD3 of Cluster 8, Asp27 of ApCARD interacts with Lys69, Val75, and Val77 of C2CARD, with the Val77 interaction being consistent in both simulations. Tyr24 of ApCARD also engages with several C2CARD residues—Lys69, Val65, Val75, Val77, and Asp58. Interestingly, none of the C2CARD residues interacting with Tyr24 in Cluster 8 are involved in similar interactions in the Cluster 5 complex.

To summarize, although there is some degree of stability occurring during the triplicate simulations of the cross-docked complexes, the level of stability and the free energies of interaction clearly illustrate the selectivity of the ApCARD-C9CARD interaction in comparison with the cross-docked complexes.

### 3.6 Comparison of Native CARD–CARD Interactions with Cross-Dockings

The apoptosome is an oligomeric complex critical for apoptosis, where the CARD–CARD interactions between Apaf-1 and procaspase-9 form a helical assembly. This interaction recruits and activates Caspase-9, triggering the apoptotic cascade. The structure of the CARD disk in the apoptosome consists of three 1:1 protomers of Apaf-1, creating a novel helical configuration on a heptameric platform.^13,14^

Similarly, the PIDDosome also relies on CARD–CARD interactions for caspase recruitment, but in this case, the CARD domain of RAIDD recruits Caspase-2. The PIDDosome’s core is composed of five PIDD DD molecules and seven RAIDD DD molecules, assembling into a large oligomeric structure. While the exact structure of the CARD disk in the PIDDosome remains unresolved, it is thought to adopt a spiral conformation, analogous to the apoptosome, facilitating Caspase-2 activation through proximity-induced dimerization.^74−76^

The structural similarities in the assembly of the apoptosome and PIDDosome complexes prompted us to investigate potential cross-interactions between their CARD domains. Specifically, we performed cross-docking simulations of the CARD domains from each complex to explore how the interaction modes might differ when attempting to bind biologically incompatible CARDs. This approach allowed us to examine structural and binding dynamics unique to each complex, offering insights into how CARD specificity may be dictated by subtle structural and electrostatic differences between the two assemblies.

Comparing the results obtained from the MD simulations of the ApCARD-C9CARD complex to the cross-dockings of ApCARD-C2CARD and RdCARD-C9CARD, it is clear that the natural CARD–CARD interaction within the apoptosome is much more stable than the cross-docking complexes (Table 1). This is not surprising given that the latter CARD domains are not expected to interact in a biological setting.

**Table 1:** Comparison of MD Simulation Results for Native CARD–CARD Interactions and CARD–CARD Cross-Dockings

Type	Complex	Apoptosome	PIDDosome	RMSD (Å)	MMGBSA(kcal/mol)
Native CARD–CARD interaction	ApCARD -C9CARD	ApCARD C9CARD		1.41 ± 0.22	-61.41 ± 7.81
CARD–CARD cross-docking	RdCARD–C9CARD	C9CARD	RdCARD	10.35 ± 4.28	-45.98 ± 19.24
	ApCARD -C2CARD Cluster 5	ApCARD	C2CARD	8.88 ± 3.71	-36.50 ± 12.85
	ApCARD -C2CARD Cluster 8	ApCARD	C2CARD	8.25 ± 4.95	-23.17 ± 12.92

The average RMSD values for the ApCARD-C9CARD is 1.41 Å with very small fluctuations, whereas, for all the cross-docked systems, the RMSD mean values are greater than 8 Å, and several of the simulation trajectories were clearly dissociative.

The high stability and strong interaction of the ApCARD-C9CARD complexes are also seen in the MMGBSA values. In contrast, the cross-docked complexes exhibit significantly reduced interaction strength and a much larger variance in the data, likely due to the dissociative behavior observed in several trajectories.

The CARD structure is highly conserved in CARD-containing proteins, with complementary charged surfaces essential for the CARD–CARD interaction,^39,77,78^ The charge distribution and topology of the domain surfaces determine the binding specificity between different CARDs.^39^ The native ApCARD-C9CARD pair exhibits a distinct electrostatic potential distribution at its type I interface, as described above and depicted in Figure 2C, with ApCARD predominantly negatively charged and C9CARD predominantly positively charged. This charge complementarity strongly favors the interaction between these two domains, leading to a highly stable complex with the lowest recorded interaction energy of −61.41 ± 7.81 kcal/mol.

**Figure 9:**
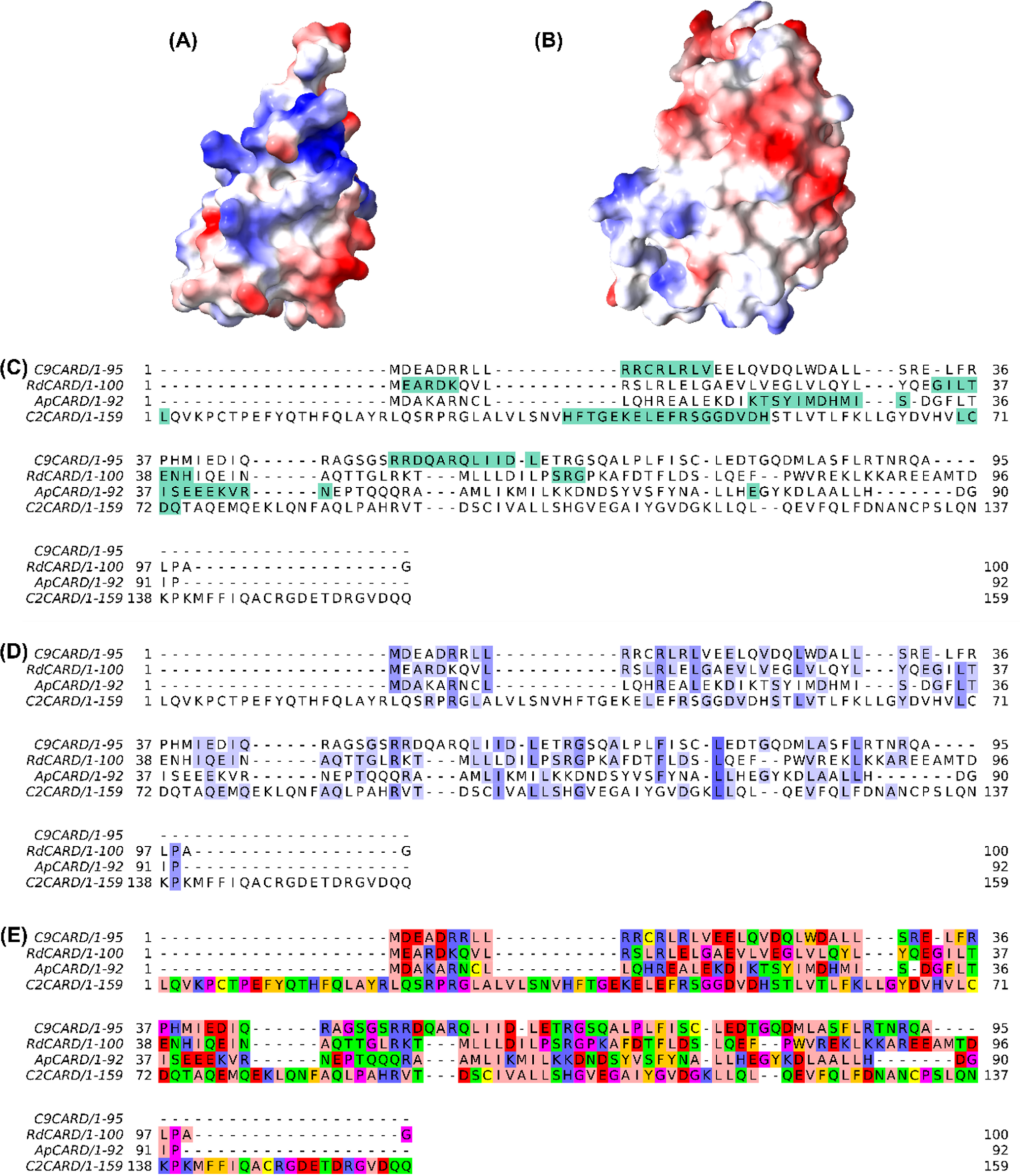
Multiple sequence alignment of four CARD domains and analysis of CARD domain interacting regions. (A, B) Representation of the protein electrostatic potential surface of (A) RdCARD and (B) C2CARD; (C) Interacting regions in native CARD pairs are highlighted in green; (D) Highlighted regions present conserved physicochemical properties, with conservation index indicated by shading intensity; (E) Residues are colored according to the Zappo scheme, which groups amino acids by physicochemical properties: aliphatic/hydrophobic (pink), aromatic (orange), positive (blue), negative (red), hydrophilic (green), conformationally special (magenta), and cysteine (yellow).

In contrast, the charged patches on the interacting interfaces of RdCARD and C2CARD are more sparsely distributed, indicating that their interaction is likely guided by specific charge patterns rather than broad charge complementarity. Interestingly, the interaction interface of RdCARD is predominantly negatively charged (Figure 9A), while C2CARD has a predominantly positive charge (Figure 9B). Previous studies suggest that both ApCARD and C2CARD function similarly, complementing the basic regions of their respective binding partners.^14^ Therefore, it is unsurprising that attempting to bind ApCARD and C2CARD—both featuring acidic surfaces—yields unstable interactions.

However, the cross-docked structures exhibited significant MMGBSA energy fluctuations throughout the MD simulations, likely due to their instability. This instability may stem from the structures encountering charged patches as they deviate from their original docked conformations, where binding is energetically more favorable. This could explain the greater variability in energy values observed in the cross-docking simulations compared to the ApCARD-C9CARD interaction.^41^

We performed a multiple sequence alignment of the four CARDs to analyze the complementarity of the interacting regions. The global alignment in the Figure 9C highlights the interacting regions in the CARD domains, revealing partial conservation in the interacting regions of the two caspase CARD domains. This suggests that these regions have maintained a similar sequence across different CARD domains, which is important for their functional interaction. However, the other interacting regions do not align well across the domains, indicating variability in sequence composition outside these conserved sites.

Interestingly, despite Caspase-2 being recognized as the most evolutionarily conserved caspase,^79^ C2CARD exhibits the lowest sequence identity compared to the other three CARD domains in this study (see Table S7). In contrast, RdCARD and C9CARD demonstrate the highest sequence identity, with a value of 0.247. We further examined sequence conservation according to the physicochemical properties of amino acids, as shown in Figure 9D. This representation highlights regions of significant concordance across the CARD domains, suggesting that conserved residues likely share similar physicochemical properties—such as charge, hydrophobicity, or polarity—indicating their potential structural or functional importance.

To further explore the physicochemical characteristics of the amino acids, we applied the Zappo scheme, categorizing residues into groups based on their properties (Figures 9E and S9). Analysis of these groups within the CARD sequences revealed that hydrophobic residues are most abundant across the four domains, likely contributing to the stabilization of the three-dimensional structure by orienting toward the interior. Notably, C2CARD contains the highest proportion of hydrophilic residues, while C9CARD shows an abundance of positively charged residues, contributing to its positively charged surface that facilitates interactions with ApCARD.

## 4 Conclusion

In this study, we employed computational approaches to analyze the interaction between the CARD domains of two proteins, Apaf-1 and Caspase-9, key to apoptotic signaling initiated through the apoptosome. Our results highlight the significance of the stability of the interaction between CARD domains, which enables apoptotic signaling to be carried forward. Although experimental characterization of CARD–CARD interactions within the apoptosome is available, our analysis provides dynamic information, adding detail on key residues and interaction types specific to the ApCARD-C9CARD pair. We identified a list of critical residues involved in the interaction between ApCARD and C9CARD and compared our findings with existing experimental data.

Key interacting pairs in ApCARD (Tyr24, Asp27, Ile30, Ser31 in α-helix H2, and Ile37, Glu40 in H3) and C9CARD (Arg10, Arg13, Leu14 in H1, and Arg52, Arg56, Ile60 in H4) demonstrated consistent stability throughout the simulations. Notably, Asp27 in ApCARD forms a stable salt bridge with Arg56 in C9CARD, persisting across 300 ns, while Glu40 in ApCARD consistently interacts with Arg13. The hydrophobic interactions between Ile30 in ApCARD and Arg10/Arg13 in C9CARD also remained highly stable. Alanine scanning confirmed these residues as crucial contributors to the interaction energy.

We also validated the meta-approach for protein–protein docking^56^ by correctly predicting the ApCARD-C9CARD interaction, and used the same approach followed by MD simulations to analyze the selectivity of the CARD domains in this pair. As part of this validation, we ensured that the meta-docking approach did not impose interactions between domains that do not biologically interact, confirming that it is not subject to bias. This was done by exploring the potential complexes and interactions involving the CARD domains from the apoptosome and the PIDDosome (RAIDD and Caspase 2 CARDs). Our analysis revealed that the native interaction between the CARD domains is significantly more stable than the cross-docking complexes, highlighting the importance of specificity in interactions between different CARD domains across distinct protein complexes.

Sequence alignment of the four CARDs showed that while the interacting regions are generally conserved, the specific interfaces do not rely on structural similarity for complementarity. The ApCARD-C9CARD pair, with its distinct charge complementarity—ApCARD being negatively charged and C9CARD positively charged—forms a highly stable complex with the lowest interaction energy. In contrast, the more balanced charge distribution in RdCARD and C2CARD suggests their interaction is driven by specific charge patterns, explaining the greater variability in energy values during cross-docking simulations.

In conclusion, protein docking coupled with simulations provides valuable insights into the molecular mechanisms of protein–protein interactions involving the CARD domains in the apoptosome. The specificity and stability of the interactions can be detailed at very high level and has the potential for future therapeutic interventions targeting related pathways or complexes.

## Data Availability

Docked protein structures and MD simulation trajectories are available freely for download via Zenodo.org: doi/10.5281/zenodo.13736242.

## Supporting Information Available

ApCARD-C9CARD crystal structures; in-depth analyses of amino acid pair interactions, RMSD data and MMGBSA from the different MD simulations of ApCARD-C9CARD, RdCARD-C9CARD and ApCARD-C2CARD; and analyses of sequence identities and amino acid property types of the four CARD domains (PDF)
