# Correction to “Computational Characterization of the Interaction of CARD Domains in the Apoptosome”

**DOI:** 10.1021/acs.biochem.5c00490

**Published:** 2025-09-16

**Authors:** Rita Ortega-Vallbona, Linda Johansson, Laureano E. Carpio, Eva Serrano-Candelas, Sayyed Jalil Mahdizadeh, Howard Fearnhead, Rafael Gozalbes, Leif A. Eriksson

The article describes the interaction between the CARD domains of APAF-1 and Caspase 9. As a hypothesis of CARD domain specificity, and a minor part of the study, cross-docking is also performed toward the interacting CARD domains of the PIDDosome, i.e., RAIDD-CARD and Caspase 2 CARD.

Due to human error, the domain described in the orginal article as the Caspase 2 CARD domain (C2CARD) is unfortunately incorrect. While being a domain of Caspase 2, the data do not describe the CARD domain.

Section 3.5, Figures 7 and 8, and part of Section 3.6 describing C2CARD or discussing cross-docking between ApCARD and C2CARD, shall thus be disregarded.

Corrected versions of Table 1 and Figure 9 are provided here.

**Table 1:** Comparison of MD Simulation Results for Native CARD–CARD Interactions and CARD–CARD Cross-Dockings

Type	Complex	Apoptosome	PIDDosome	RMSD (Å)	MMGBSA (kcal/mol)
Native CARD–CARD interaction	ApCARD -C9CARD	ApCARD C9CARD		1.41 ± 0.22	–61.41 ± 7.81
CARD–CARD cross-docking	RdCARD - C9CARD	C9CARD	RdCARD	10.35 ± 4.28	–45.98 ± 19.24

**Figure 9:**
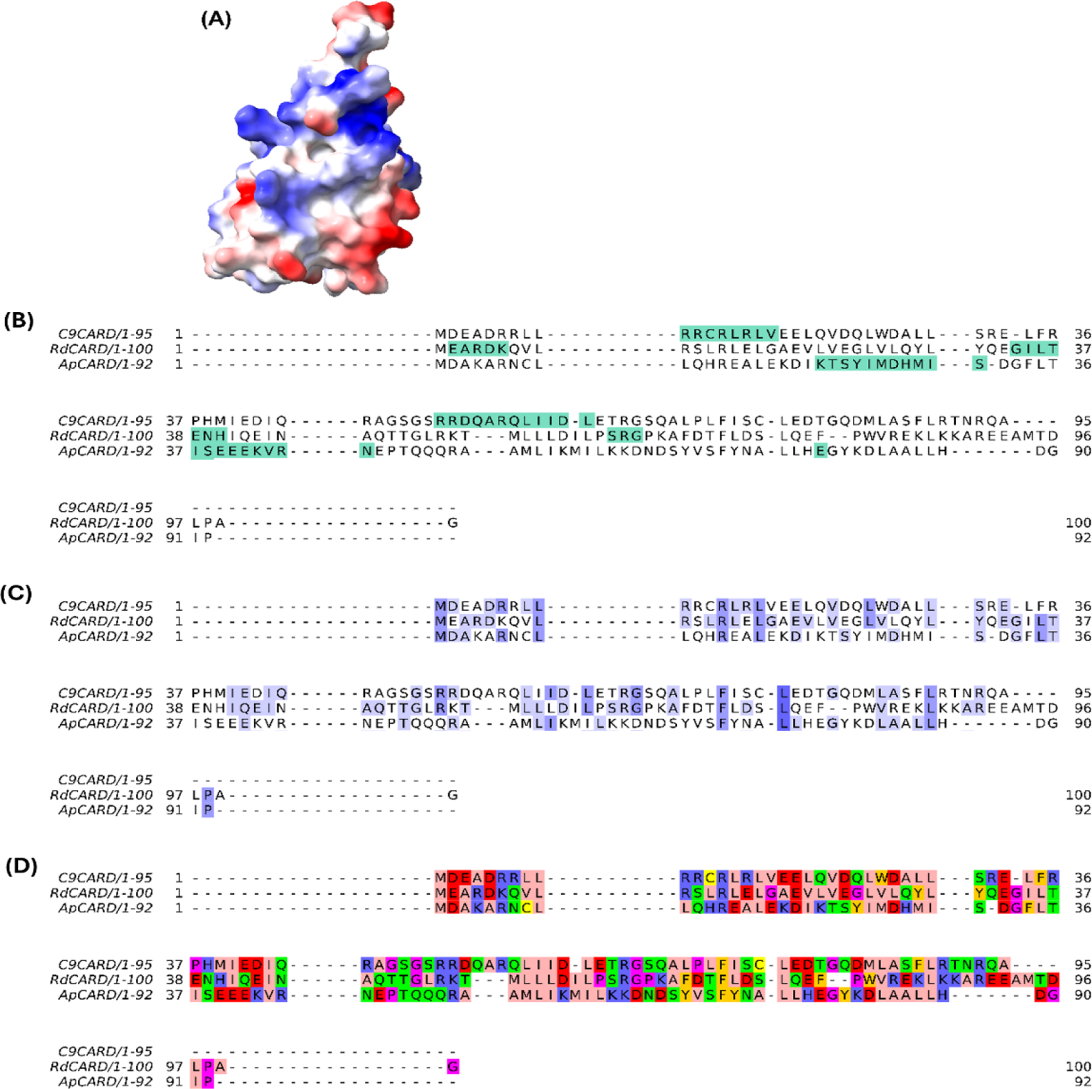
Multiple sequence alignment of three CARD domains and analysis of CARD domain interacting regions. (A) Representation of the protein electrostatic potential surface of RdCARD. (B) Interacting regions in native CARD pairs are highlighted in green. (C) Highlighted regions present conserved physicochemical properties, with conservation index indicated by shading intensity. (D) Residues are colored according to the Zappo scheme, which groups amino acids by physicochemical properties: aliphatic/hydrophobic (pink), aromatic (orange), positive (blue), negative (red), hydrophilic (green), conformationally special (magenta), and cysteine (yellow).

## Supplementary Material



## Notes

The Supporting Information is corrected accordingly and contains information about the available ApCARD-C9CARD crystal structures; in-depth analyses of amino acid pair interactions, RMSD data and MMGBSA from the different MD simulations of ApCARD-C9CARD and RdCARD-C9CARD; and analyses of sequence identities and amino acid property types of the three CARD domains. (PDF)

