# Supplementary material for: Computational Characterization of the Interaction of CARD Domains in the Apoptosome
Source: Biochemistry. 2025 Jan 6;64(2):401–18. doi: 10.1021/acs.biochem.4c00583 (PMC11755718; doi:10.1021/acs.biochem.4c00583)
Supplement: Supplementary file 1 — bi4c00583_si_001.pdf [file bi4c00583_si_001.pdf]

SUPPLEMENTARY INFORMATION

Computational characterisation of the interaction of CARD domains in the apoptosome

Rita Ortega-Vallbona[1], Linda Johansson[2], Laureano E. Carpio[1,3], Eva Serrano-Candelas[1], Sayyed Jalil Mahdizadeh[2], Howard Fearnhead[4], Rafael Gozalbes[1,3], Leif A. Eriksson[2]*.

[1]ProtoQSAR SL, Centro Europeo de Empresas Innovadoras, Parque Tecnológico de Valencia, 46980 Paterna (Valencia), Spain

[2]Department of Chemistry and Molecular Biology, University of Gothenburg, 405 30 Göteborg, Sweden.

[3]Moldrug AI Systems SL, Olimpia Arozena Torres 45, 46018 Valencia, Spain

[4]Pharmacology and Therapeutics, National University of Ireland Galway, Galway, Ireland, H91 TK33

*Corresponding Author: Leif A. Eriksson.
Email: leif.eriksson@chem.gu.se

TABLE OF CONTENTS

Table S1. Information about the six crystals structures showing ApCARD-C9CARD interactions. S3

Table S2. Interaction in Type 1 interface between ApCARD and C9CARD of crystal structure 4RHW. S5

Table S3. RMSD (Å) and MMGBSA binding energies (kcal/mol) for molecular dynamics simulations of CARD domain pairs. S6

Table S4. Pairwise statistical comparisons of RMSD values across molecular dynamics simulations of CARD domain pairs. S7

Table S5. Pairwise statistical comparisons of MMGBSA binding free energies across molecular dynamics simulations of CARD domain pairs. S10

Figure S1. Analysis of interactions of the ApCARD-C9CARD complex at the type I interface as a function of time. S13

Table S6. Pairwise interactions in the three MD simulations performed with ApCARD and C9CARD. S14

Figure S2. Kelley penalty plots for optimal clustering in meta-docking analyses of CARD domain pairs. S15

Figure S3. Heatmap showing the number of interactions by pair of residues as a function of time for MD simulation 1 of the cross-docked RdCARD-C9CARD complex. S16

Figure S4. Heatmaps showing the number of interactions by pair of residues as a function of time for MD simulations 2 and 3 of the cross-docked RdCARD-C9CARD complex.S17

Figure S5. Superposed structure of the RdCARD-C9CARD complex at the start and end of the MD1 simulation. S18

Figure S6. Superposed structure of the ApCARD-C2CARD Cluster 5 complex at the start and end of the MD3 simulation. S19

Figure S7. Heatmaps showing the number of interactions by pair of residues as a function of time for the MD simulations of the cross-docked ApCARD-C2CARD Cluster 5 complex. S20

Figure S8. Heatmaps showing the number of interactions by pair of residues as a function of time for the MD simulations of the cross-docked ApCARD-C2CARD Cluster 8 complex. S21

Table S7. Sequence identities of the four CARD domains. S22

Figure S9. Counts of amino acids by types in the four CARD domains, following the Zappo classification. S22

References (for Table S1) S23

Table S1. Information about the six crystals presenting ApCARD-C9CARD interactions. Types of interfaces described in the publications associated to each published structure and important residues that have been experimentally characterized.

PDB ID	Year	Resolution (Å)	Interfaces	Residues important for type I interface	Ref.
**3YGS**	2000	2.50	Type I: (only one considered) positively charged helices H1a/H1b and H4 of C9CARD recognized by negatively charged helices H2 and H3 of ApCARD.	ApCARD: two mutations, Asp27Ala on helix H2 and Glu40Ala on helix H3, eliminated interaction with caspase-9. Ser31Ala mutation weakened but failed to abolish interaction.<br>C9CARD: two mutations, Arg13Ala at the predicted hinge region between H1a and H1b helices and Arg56Ala on helix H4, prevented interaction with wild-type Apaf-1 CARD. Two additional mutations, Arg11Ala at the end of helix H1 and Arg52Ala on helix H4, also significantly reduced the interaction.	[1]
**3J2T**	2013	9.50	Not described in publication. Acentric CARD-CARD disc is not shown in the crystal.	Interacting residues are not described in the publication	[2]
**4RHW**	2014	2.10	Type I: C9CARD uses two α-helices H1/H4 to closely stack against helices H2/H3 from an ApCARD (corresponds to 3YGS).<br>Type II: C9CARD H4 and interhelical loops L23/L45 to contact H6 and loops L12/L56 from the other ApCARD.<br>Type III: between ApCARD domains- H3	Important interdomain hydrogen bonds: between Glu40 of ApCARD and Arg13 of C9CARD and between Asp27 of ApCARD and Arg52 of C9CARD.	[3]

			from one and L34/H4 from the other.		
**5JUY**	2016	4.10	Agree with 4RHW. Type II interfaces between color-coded CARD pairs.	They don’t describe any additional key residues in type I interface.	[4]
**5WVC**	2017	2.99	One protomer is formed by one C9CARD and one ApCARD joined by type I interface.<br>Protomers assemble through a type II and two type III interfaces, and one type I-like interface to complete the helical turn type II -III - III interfaces form a unique surface of interaction between protomers.	They compare their interacting residues of Caspase9 to 4RHW and 5JUY. They find conserved interacting residues of C9 in type I interface.	[5]
**5WVE**	2017	4.40	Type I: helices H2 and H3 of ApCARD interact with H1 and H4 of C9CARD.<br>Type II: intervening turns between H1-H2 and H5-H6 of ApCARD and turns between H2-H3 and H4-H5 of C9CARD.	Resolution of structure is insufficient for accurate assignment of specific interactions involving amino acid side chains in the CARD complex.	[6]

Table S2. Interactions at the type I interface between chain B (ApCARD) and chain E (C9CARD) in the crystal structure of the 4RHW complex. The table details the interacting residues from ApCARD and C9CARD, the distance between them (in Å), and the specific interaction types (e.g., hydrogen bonds, salt bridges, or hydrophobic contacts).

Residues ApCARD	Residues C9CARD	Distance(A)	Specific Interactions
B:GLU 78	E:ARG 52	3.3	
B:GLU 78	E:LEU 14	3.8	
B:ARG 44	E:ARG 56	2.2	
B:ARG 44	E:ILE 60	1.9	
B:ARG 44	E:GLN 57	2.7	
B:ARG 44	E:ARG 13	3.8	
B:ARG 44	E:ALA 46	3.7	
B:GLU 40	E:ARG 13	2.1	2x hb, 1x salt bridge to E:ARG 13
B:GLU 40	E:ARG 56	3.1	
B:GLU 40	E:ILE 60	3.2	
B:ILE 37	E:ARG 10	1.8	
B:ILE 37	E:ILE 60	2.2	
B:ILE 37	E:ARG 13	2.4	
B:ILE 37	E:GLU 63	3.1	
B:SER 31	E:ARG 13	2.1	
B:SER 31	E:ARG 10	2.4	
B:SER 31	E:ARG 11	2.6	
B:SER 31	E:LEU 14	2.2	
B:ILE 30	E:ARG 10	2.3	
B:ILE 30	E:ARG 13	2.4	
B:HIE 28	E:LEU 14	2.2	
B:ASP 27	E:LEU 14	2.6	
B:ASP 27	E:ARG 13	2.2	1x hb to E:ARG 13
B:ASP 27	E:VAL 17	2.6	
B:ASP 27	E:ARG 52	2.1	1x hb, 1x salt bridge to E:ARG 52
B:ASP 27	E:ARG 56	2.0	2x hb, 1x salt bridge to E:ARG 56
B:TYR 24	E:ARG 56	2.9	
B:TYR 24	E:ARG 52	2.3	
B:SER 23	E:ARG 56	2.7	
B:SER 23	E:ASP 53	3.8	
B:LYS 21	E:ASP 53	3.9	

Table S3. RMSD (Å) and MMGBSA binding energies (kcal/mol) for molecular dynamics simulations of CARD domain pairs. Mean, standard deviation (SD), and median values are provided for ApCARD-C9CARD, RdCARD-C9CARD, and ApCARD-C2CARD (clusters 5 and 8) across individual (MD1, MD2, MD3) and combined simulations (all).

	RMSD (Å)			MMGBSA (kcal/mol)		
**Sample**	**Mean**	**SD**	**Median**	**Mean**	**SD**	**Median**
ApCARD-C9CARD MD1	1,46	0,19	1,45	-62,44	6,97	-62,12
ApCARD-C9CARD MD2	1,39	0,27	1,37	-64,21	8,07	-63,42
ApCARD-C9CARD MD3	1,38	0,19	1,36	-57,57	6,74	-56,91
ApCARD-C9CARD all	*1,41*	*0,22*	*1,40*	*-61,41*	*7,81*	*-60,68*
RdCARD-C9CARD MD1	5,38	0,47	5,31	-62,08	11,85	-62,45
RdCARD-C9CARD MD2	14,32	2,71	15,51	-26,90	10,58	-24,16
RdCARD-C9CARD MD3	11,33	2,43	12,57	-48,95	15,02	-50,18
RdCARD-C9CARD all	*10,35*	*4,28*	*11,20*	*-45,98*	*19,24*	*-45,93*
ApCARD-C2CARD cluster5 MD1	8,82	1,13	9,16	-48,70	8,70	-49,64
ApCARD-C2CARD cluster5 MD2	13,23	1,56	13,23	-36,04	10,02	-38,26
ApCARD-C2CARD cluster5 MD3	4,59	0,40	4,60	-24,76	5,69	-24,71
ApCARD-C2CARD cluster5 all	*8,88*	*3,71*	*9,12*	*-36,50*	*12,85*	*-36,61*
ApCARD-C2CARD cluster8 MD1	8,60	7,58	4,25	-19,97	15,76	-20,91
ApCARD-C2CARD cluster8 MD2	9,79	2,63	10,95	-27,17	10,58	-29,25
ApCARD-C2CARD cluster8 MD3	6,35	1,73	5,77	-22,37	10,67	-21,16
ApCARD-C2CARD cluster8 all	*8,25*	*4,95*	*7,68*	*-23,17*	*12,92*	*-24,47*

Table S4. Pairwise statistical comparisons of RMSD values across molecular dynamics simulations of CARD domain pairs, performed using the Mann-Whitney U test. Significant differences ($p < 0.05$) are indicated in the "Significant" column. Comparisons include the MD1, MD2, MD3 of ApCARD-C9CARD, RdCARD-C9CARD, and ApCARD-C2CARD clusters (5 and 8).

Sample A	Sample B	p-value	Significant
ApCARD-C9CARD MD1	ApCARD-C9CARD MD2	0,00287031	TRUE
ApCARD-C9CARD MD1	ApCARD-C9CARD MD3	9,1214E-06	TRUE
ApCARD-C9CARD MD2	ApCARD-C9CARD MD3	0,98658571	FALSE
ApCARD-C9CARD MD1	ApCARD-C2CARD cluster5 MD1	5,5494E-60	TRUE
ApCARD-C9CARD MD1	ApCARD-C2CARD cluster5 MD2	5,5494E-60	TRUE
ApCARD-C9CARD MD1	ApCARD-C2CARD cluster5 MD3	5,5494E-60	TRUE
ApCARD-C9CARD MD2	ApCARD-C2CARD cluster5 MD1	5,5494E-60	TRUE
ApCARD-C9CARD MD2	ApCARD-C2CARD cluster5 MD2	5,5494E-60	TRUE
ApCARD-C9CARD MD2	ApCARD-C2CARD cluster5 MD3	5,5494E-60	TRUE
ApCARD-C9CARD MD3	ApCARD-C2CARD cluster5 MD1	5,5494E-60	TRUE
ApCARD-C9CARD MD3	ApCARD-C2CARD cluster5 MD2	5,5494E-60	TRUE
ApCARD-C9CARD MD3	ApCARD-C2CARD cluster5 MD3	5,5494E-60	TRUE
ApCARD-C9CARD MD1	ApCARD-C2CARD cluster8 MD1	5,5494E-60	TRUE
ApCARD-C9CARD MD1	ApCARD-C2CARD cluster8 MD2	5,5494E-60	TRUE
ApCARD-C9CARD MD1	ApCARD-C2CARD cluster8 MD3	5,5494E-60	TRUE
ApCARD-C9CARD MD2	ApCARD-C2CARD cluster8 MD1	5,5494E-60	TRUE
ApCARD-C9CARD MD2	ApCARD-C2CARD cluster8 MD2	5,5494E-60	TRUE
ApCARD-C9CARD MD2	ApCARD-C2CARD cluster8 MD3	5,5494E-60	TRUE
ApCARD-C9CARD MD3	ApCARD-C2CARD cluster8 MD1	5,5494E-60	TRUE
ApCARD-C9CARD MD3	ApCARD-C2CARD cluster8 MD2	5,5494E-60	TRUE
ApCARD-C9CARD MD3	ApCARD-C2CARD cluster8 MD3	5,5494E-60	TRUE
ApCARD-C9CARD MD1	RdCARD-C9CARD MD1	5,5494E-60	TRUE
ApCARD-C9CARD MD1	RdCARD-C9CARD MD2	5,5494E-60	TRUE

Sample A	Sample B	p-value	Significant
ApCARD-C9CARD MD1	RdCARD-C9CARD MD3	5,5494E-60	TRUE
ApCARD-C9CARD MD2	RdCARD-C9CARD MD1	5,5494E-60	TRUE
ApCARD-C9CARD MD2	RdCARD-C9CARD MD2	5,5494E-60	TRUE
ApCARD-C9CARD MD2	RdCARD-C9CARD MD3	5,5494E-60	TRUE
ApCARD-C9CARD MD3	RdCARD-C9CARD MD1	5,5494E-60	TRUE
ApCARD-C9CARD MD3	RdCARD-C9CARD MD2	5,5494E-60	TRUE
ApCARD-C9CARD MD3	RdCARD-C9CARD MD3	5,5494E-60	TRUE
ApCARD-C2CARD cluster5 MD1	ApCARD-C2CARD cluster5 MD2	5,0011E-44	TRUE
ApCARD-C2CARD cluster5 MD1	ApCARD-C2CARD cluster5 MD3	7,7136E-58	TRUE
ApCARD-C2CARD cluster5 MD2	ApCARD-C2CARD cluster5 MD3	1,0308E-50	TRUE
ApCARD-C2CARD cluster5 MD1	ApCARD-C2CARD cluster8 MD1	1,6437E-06	TRUE
ApCARD-C2CARD cluster5 MD1	ApCARD-C2CARD cluster8 MD2	3,0072E-12	TRUE
ApCARD-C2CARD cluster5 MD1	ApCARD-C2CARD cluster8 MD3	6,889E-27	TRUE
ApCARD-C2CARD cluster5 MD2	ApCARD-C2CARD cluster8 MD1	1,6612E-07	TRUE
ApCARD-C2CARD cluster5 MD2	ApCARD-C2CARD cluster8 MD2	2,2647E-43	TRUE
ApCARD-C2CARD cluster5 MD2	ApCARD-C2CARD cluster8 MD3	3,8845E-48	TRUE
ApCARD-C2CARD cluster5 MD3	ApCARD-C2CARD cluster8 MD1	0,02673098	TRUE
ApCARD-C2CARD cluster5 MD3	ApCARD-C2CARD cluster8 MD2	4,2722E-24	TRUE
ApCARD-C2CARD cluster5 MD3	ApCARD-C2CARD cluster8 MD3	2,7123E-23	TRUE
ApCARD-C2CARD cluster5 MD1	RdCARD-C9CARD MD1	2,8767E-48	TRUE
ApCARD-C2CARD cluster5 MD1	RdCARD-C9CARD MD2	9,0646E-43	TRUE
ApCARD-C2CARD cluster5 MD1	RdCARD-C9CARD MD3	3,6609E-16	TRUE
ApCARD-C2CARD cluster5 MD2	RdCARD-C9CARD MD1	1,8876E-50	TRUE
ApCARD-C2CARD cluster5 MD2	RdCARD-C9CARD MD2	1,9605E-06	TRUE
ApCARD-C2CARD cluster5 MD2	RdCARD-C9CARD MD3	9,6477E-09	TRUE
ApCARD-C2CARD cluster5 MD3	RdCARD-C9CARD MD1	3,1345E-38	TRUE
ApCARD-C2CARD cluster5 MD3	RdCARD-C9CARD MD2	2,8504E-46	TRUE
ApCARD-C2CARD cluster5 MD3	RdCARD-C9CARD MD3	6,6592E-59	TRUE

Sample A	Sample B	p-value	Significant
ApCARD-C2CARD cluster8 MD1	ApCARD-C2CARD cluster8 MD2	0,00046458	TRUE
ApCARD-C2CARD cluster8 MD1	ApCARD-C2CARD cluster8 MD3	0,00020068	TRUE
ApCARD-C2CARD cluster8 MD2	ApCARD-C2CARD cluster8 MD3	1,0119E-21	TRUE
ApCARD-C2CARD cluster8 MD1	RdCARD-C9CARD MD1	1,3986E-05	TRUE
ApCARD-C2CARD cluster8 MD1	RdCARD-C9CARD MD2	3,3397E-09	TRUE
ApCARD-C2CARD cluster8 MD1	RdCARD-C9CARD MD3	3,4296E-07	TRUE
ApCARD-C2CARD cluster8 MD2	RdCARD-C9CARD MD1	6,1833E-23	TRUE
ApCARD-C2CARD cluster8 MD2	RdCARD-C9CARD MD2	1,479E-33	TRUE
ApCARD-C2CARD cluster8 MD2	RdCARD-C9CARD MD3	1,3208E-05	TRUE
ApCARD-C2CARD cluster8 MD3	RdCARD-C9CARD MD1	0,00779276	TRUE
ApCARD-C2CARD cluster8 MD3	RdCARD-C9CARD MD2	5,4005E-44	TRUE
ApCARD-C2CARD cluster8 MD3	RdCARD-C9CARD MD3	9,5135E-37	TRUE
RdCARD-C9CARD MD1	RdCARD-C9CARD MD2	7,9131E-44	TRUE
RdCARD-C9CARD MD1	RdCARD-C9CARD MD3	3,0328E-50	TRUE
RdCARD-C9CARD MD2	RdCARD-C9CARD MD3	3,331E-16	TRUE

Table S5. Pairwise statistical comparisons of MMGBSA binding free energies across molecular dynamics simulations of CARD domain pairs, performed using the Mann-Whitney U test. Significant differences ($p < 0.05$) are indicated in the "Significant" column.Comparisons include the MD1, MD2, MD3 of ApCARD-C9CARD, RdCARD-C9CARD, and ApCARD-C2CARD clusters (5 and 8).

Sample A	Sample B	p-value	Significant
ApCARD-C9CARD MD1	ApCARD-C9CARD MD2	0,11114923	FALSE
ApCARD-C9CARD MD1	ApCARD-C9CARD MD3	5,1917E-07	TRUE
ApCARD-C9CARD MD2	ApCARD-C9CARD MD3	3,2353E-09	TRUE
ApCARD-C9CARD MD1	ApCARD-C2CARD cluster5 MD1	1,0688E-27	TRUE
ApCARD-C9CARD MD1	ApCARD-C2CARD cluster5 MD2	5,0955E-54	TRUE
ApCARD-C9CARD MD1	ApCARD-C2CARD cluster5 MD3	1,1099E-59	TRUE
ApCARD-C9CARD MD2	ApCARD-C2CARD cluster5 MD1	5,5633E-30	TRUE
ApCARD-C9CARD MD2	ApCARD-C2CARD cluster5 MD2	3,586E-54	TRUE
ApCARD-C9CARD MD2	ApCARD-C2CARD cluster5 MD3	1,1099E-59	TRUE
ApCARD-C9CARD MD3	ApCARD-C2CARD cluster5 MD1	7,6223E-13	TRUE
ApCARD-C9CARD MD3	ApCARD-C2CARD cluster5 MD2	6,3689E-48	TRUE
ApCARD-C9CARD MD3	ApCARD-C2CARD cluster5 MD3	2,2197E-59	TRUE
ApCARD-C9CARD MD1	ApCARD-C2CARD cluster8 MD1	3,7958E-57	TRUE
ApCARD-C9CARD MD1	ApCARD-C2CARD cluster8 MD2	3,8846E-59	TRUE
ApCARD-C9CARD MD1	ApCARD-C2CARD cluster8 MD3	1,0821E-57	TRUE
ApCARD-C9CARD MD2	ApCARD-C2CARD cluster8 MD1	5,0777E-57	TRUE
ApCARD-C9CARD MD2	ApCARD-C2CARD cluster8 MD2	6,6592E-59	TRUE
ApCARD-C9CARD MD2	ApCARD-C2CARD cluster8 MD3	2,0699E-57	TRUE
ApCARD-C9CARD MD3	ApCARD-C2CARD cluster8 MD1	1,7373E-54	TRUE
ApCARD-C9CARD MD3	ApCARD-C2CARD cluster8 MD2	1,6648E-58	TRUE
ApCARD-C9CARD MD3	ApCARD-C2CARD cluster8 MD3	2,0884E-54	TRUE
ApCARD-C9CARD MD1	RdCARD-C9CARD MD1	0,88920467	FALSE
ApCARD-C9CARD MD1	RdCARD-C9CARD MD2	1,0086E-53	TRUE

Sample A	Sample B	p-value	Significant
ApCARD-C9CARD MD1	RdCARD-C9CARD MD3	3,0217E-10	TRUE
ApCARD-C9CARD MD2	RdCARD-C9CARD MD1	0,20247462	FALSE
ApCARD-C9CARD MD2	RdCARD-C9CARD MD2	8,5234E-54	TRUE
ApCARD-C9CARD MD2	RdCARD-C9CARD MD3	4,434E-13	TRUE
ApCARD-C9CARD MD3	RdCARD-C9CARD MD1	0,00094788	TRUE
ApCARD-C9CARD MD3	RdCARD-C9CARD MD2	1,564E-48	TRUE
ApCARD-C9CARD MD3	RdCARD-C9CARD MD3	0,00023921	TRUE
ApCARD-C2CARD cluster5 MD1	ApCARD-C2CARD cluster5 MD2	7,0474E-20	TRUE
ApCARD-C2CARD cluster5 MD1	ApCARD-C2CARD cluster5 MD3	1,6797E-44	TRUE
ApCARD-C2CARD cluster5 MD2	ApCARD-C2CARD cluster5 MD3	5,1866E-20	TRUE
ApCARD-C2CARD cluster5 MD1	ApCARD-C2CARD cluster8 MD1	2,7827E-36	TRUE
ApCARD-C2CARD cluster5 MD1	ApCARD-C2CARD cluster8 MD2	5,2458E-36	TRUE
ApCARD-C2CARD cluster5 MD1	ApCARD-C2CARD cluster8 MD3	7,1723E-40	TRUE
ApCARD-C2CARD cluster5 MD2	ApCARD-C2CARD cluster8 MD1	9,687E-14	TRUE
ApCARD-C2CARD cluster5 MD2	ApCARD-C2CARD cluster8 MD2	2,0367E-10	TRUE
ApCARD-C2CARD cluster5 MD2	ApCARD-C2CARD cluster8 MD3	1,202E-17	TRUE
ApCARD-C2CARD cluster5 MD3	ApCARD-C2CARD cluster8 MD1	0,02897271	TRUE
ApCARD-C2CARD cluster5 MD3	ApCARD-C2CARD cluster8 MD2	0,00230754	TRUE
ApCARD-C2CARD cluster5 MD3	ApCARD-C2CARD cluster8 MD3	0,01366717	TRUE
ApCARD-C2CARD cluster5 MD1	RdCARD-C9CARD MD1	6,8924E-16	TRUE
ApCARD-C2CARD cluster5 MD1	RdCARD-C9CARD MD2	9,6017E-33	TRUE
ApCARD-C2CARD cluster5 MD1	RdCARD-C9CARD MD3	0,65327465	FALSE
ApCARD-C2CARD cluster5 MD2	RdCARD-C9CARD MD1	9,1952E-40	TRUE
ApCARD-C2CARD cluster5 MD2	RdCARD-C9CARD MD2	7,1264E-11	TRUE
ApCARD-C2CARD cluster5 MD2	RdCARD-C9CARD MD3	1,1415E-08	TRUE
ApCARD-C2CARD cluster5 MD3	RdCARD-C9CARD MD1	8,8624E-57	TRUE
ApCARD-C2CARD cluster5 MD3	RdCARD-C9CARD MD2	0,54169971	FALSE
ApCARD-C2CARD cluster5 MD3	RdCARD-C9CARD MD3	2,8662E-33	TRUE

Sample A	Sample B	p-value	Significant
ApCARD-C2CARD cluster8 MD1	ApCARD-C2CARD cluster8 MD2	0,00124109	TRUE
ApCARD-C2CARD cluster8 MD1	ApCARD-C2CARD cluster8 MD3	0,25145797	FALSE
ApCARD-C2CARD cluster8 MD2	ApCARD-C2CARD cluster8 MD3	0,00020667	TRUE
ApCARD-C2CARD cluster8 MD1	RdCARD-C9CARD MD1	5,5104E-49	TRUE
ApCARD-C2CARD cluster8 MD1	RdCARD-C9CARD MD2	0,00358312	TRUE
ApCARD-C2CARD cluster8 MD1	RdCARD-C9CARD MD3	5,7789E-26	TRUE
ApCARD-C2CARD cluster8 MD2	RdCARD-C9CARD MD1	7,9471E-53	TRUE
ApCARD-C2CARD cluster8 MD2	RdCARD-C9CARD MD2	0,28149449	FALSE
ApCARD-C2CARD cluster8 MD2	RdCARD-C9CARD MD3	4,478E-22	TRUE
ApCARD-C2CARD cluster8 MD3	RdCARD-C9CARD MD1	2,9644E-52	TRUE
ApCARD-C2CARD cluster8 MD3	RdCARD-C9CARD MD2	0,00260636	TRUE
ApCARD-C2CARD cluster8 MD3	RdCARD-C9CARD MD3	2,2259E-30	TRUE
RdCARD-C9CARD MD1	RdCARD-C9CARD MD2	3,9069E-47	TRUE
RdCARD-C9CARD MD1	RdCARD-C9CARD MD3	4,3006E-09	TRUE
RdCARD-C9CARD MD2	RdCARD-C9CARD MD3	7,2198E-24	TRUE

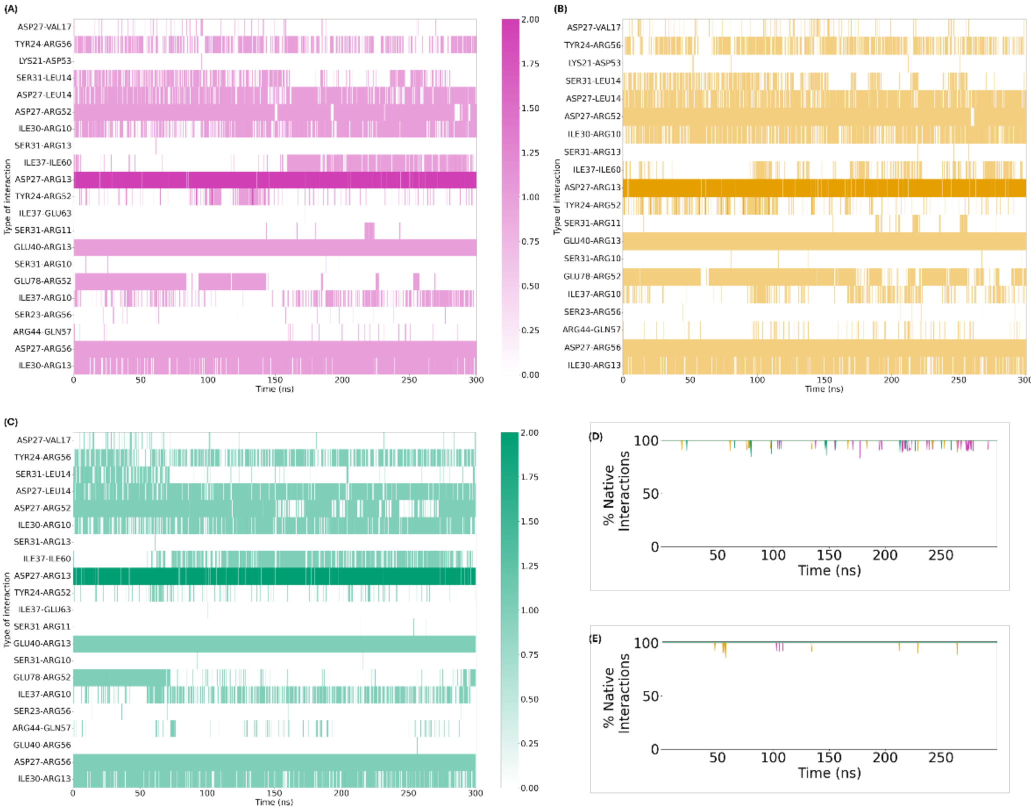

Figure S1. Analysis of interactions of the ApCARD-C9CARD complex at the type I interface as a function of time: (A, B, C) Heatmaps showing interaction counts between residue pairs over simulation time for each of the triplicate MD simulations: (A) MD1, (B) MD2, and (C) MD3; (D, E) Percentage of native interactions based on residues in (D) ApCARD and (E) C9CARD.

Table S6. Pairwise interactions in the three MD simulations performed with ApCARD and C9CARD, compared to the interactions detected in the 4RHW crystal structure.

Present in MD	Present in crystal	ApCARD	C9CARD	Specific Interactions
2	Yes	Arg 44	Arg 13	
3	Yes	Arg 44	Arg 56	
3	Yes	Arg 44	Gln 57	
2 and 3	Yes	Arg 44	Ile 60	
All 3 MDs	Yes	Asp 27	Arg 13	1x hb in all MDs and crystal
1 and 2	Yes	Asp 27	Arg 52	Non-specific; 2x hb, 1x salt bridge; 1x hb, 1x salt bridge in MD2 and crystal
All 3 MDs	Yes	Asp 27	Arg 56	2x hb, 1x salt bridge in all MDs and crystal
All 3 MDs	Yes	Asp 27	Leu 14	
All 3 MDs	Yes	Asp 27	Val 17	
All 3 MDs	Yes	Glu 40	Arg 13	2x hb, 1x salt bridge in all MDs and crystal
1 and 3	Yes	Glu 40	Arg 56	
1 and 3	Yes	Glu 40	Ile 60	
2	Yes	Glu 78	Arg 52	2x hb
2	Yes	Glu 78	Leu 14	
1	No	Gly 33	Arg 10	
2	No	His 28	Arg 52	
All 3 MDs	Yes	His 28	Leu 14	
All 3 MDs	Yes	Ile 30	Arg 10	
1 and 2	No	Ile 30	Arg 11	
All 3 MDs	Yes	Ile 30	Arg 13	
All 3 MDs	Yes	Ile 37	Arg 10	
All 3 MDs	Yes	Ile 37	Arg 13	
1 and 3	Yes	Ile 37	Glu 63	
1 and 3	Yes	Ile 37	Ile 60	
1	No	Leu 35	Arg 10	
2	No	Lys 21	Ser 48	
2	No	Met 26	Arg 13	
All 3 MDs	Yes	Ser 23	Arg 56	
All 3 MDs	Yes	Ser 31	Arg 10	
All 3 MDs	Yes	Ser 31	Arg 11	
All 3 MDs	Yes	Ser 31	Arg 13	
2	No	Ser 31	Arg 15	
All 3 MDs	Yes	Ser 31	Leu 14	
2	No	Ser 31	Leu 14	1x clash
1	No	Thr 36	Arg 10	
All 3 MDs	Yes	Tyr 24	Arg 52	

All 3 MDs	Yes	Tyr 24	Arg 56	
2	No	Tyr 24	Asp 53	1x hb
2	No	Tyr 24	Ser 50	
No	Yes	Arg 44	Ala 46	
No	Yes	Ser 23	Asp 53	

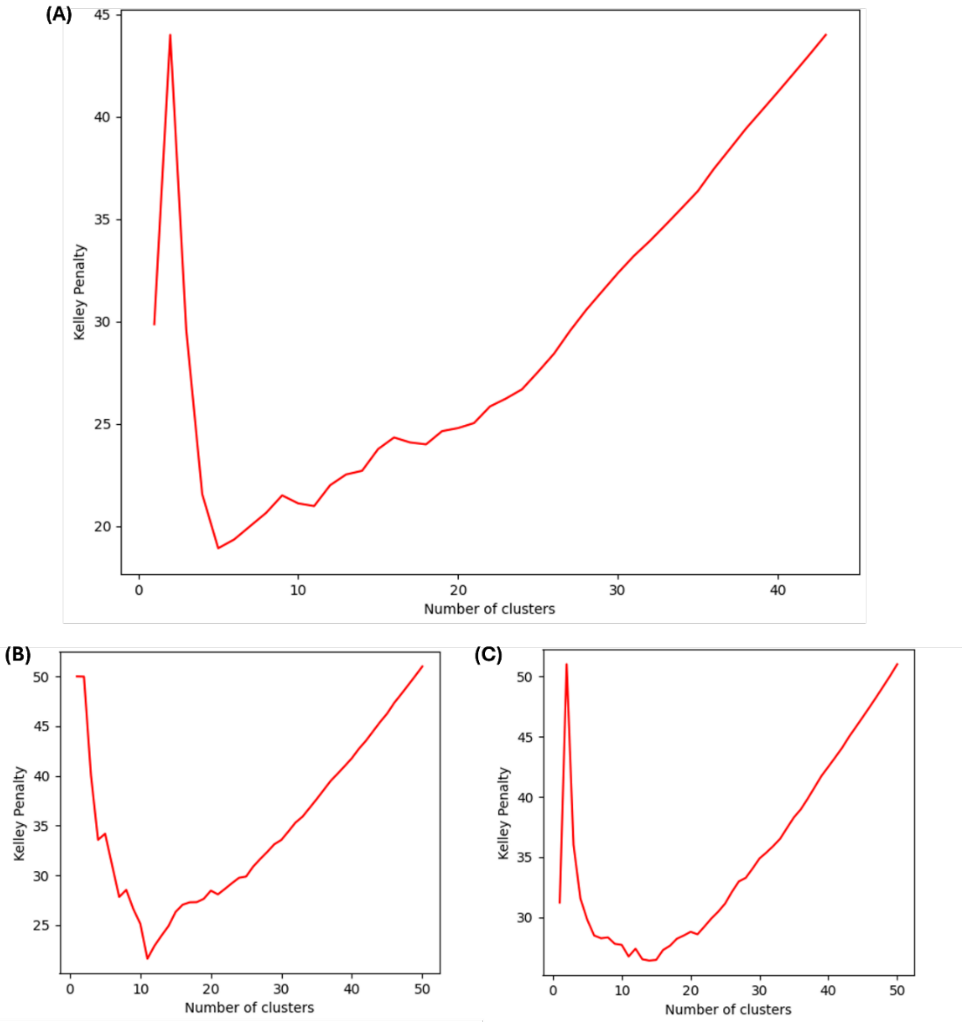

Figure S2. Kelley penalty plots for optimal clustering in meta-docking analyses of CARD domain pairs: (A) ApCARD-C9CARD, (B) RdCARD-C9CARD, and (C) ApCARD-C2CARD.

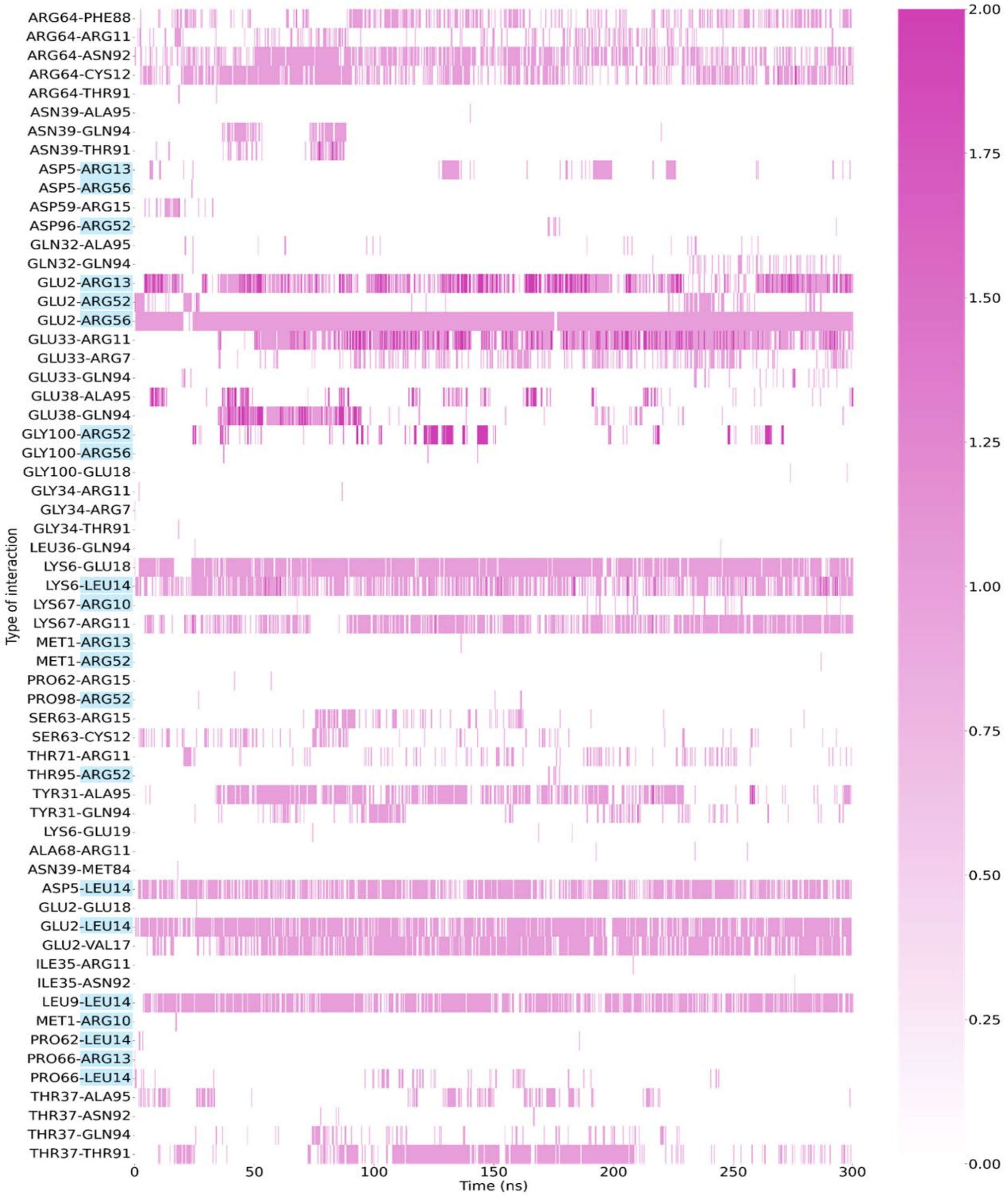

Figure S3. Heatmap showing the number of interactions by pair of residues as a function of time for MD1 of the RdCARD-C9CARD system. The residues highlighted in blue are the C9CARD residues that were identified as important for the native ApCARD-C9CARD interaction.

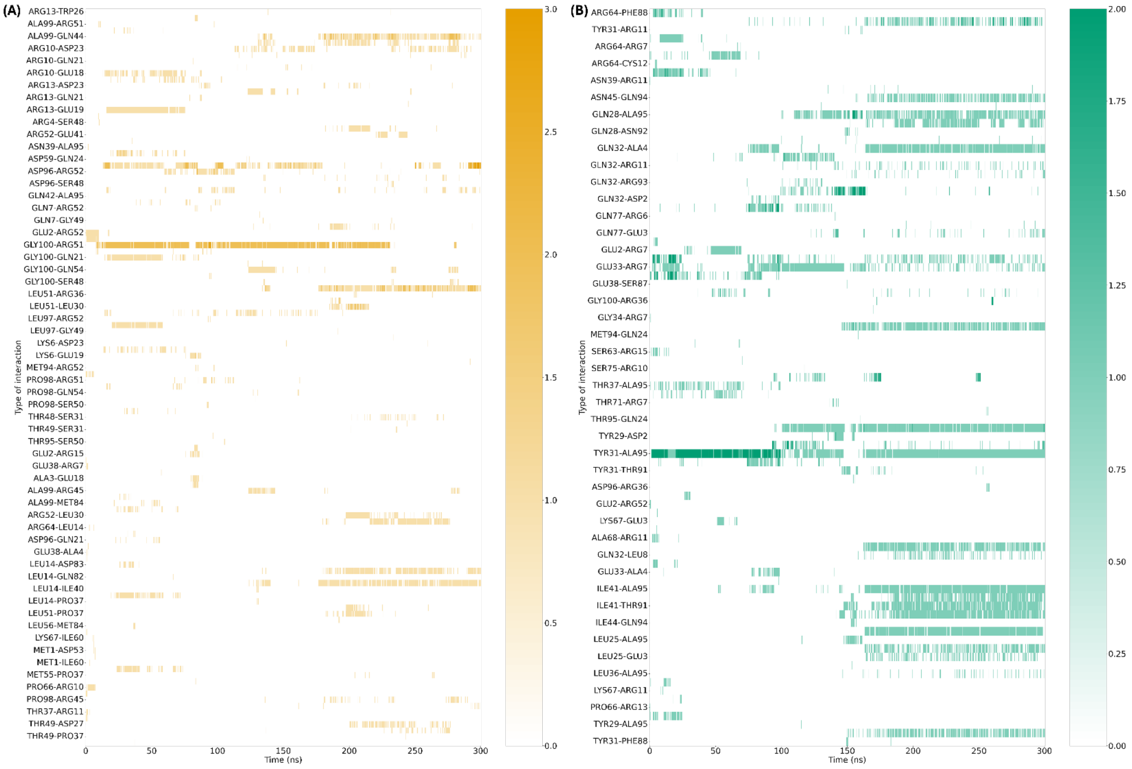

Figure S4. Heatmaps showing the number of interactions by pair of residues as a function of time for the MD2 (A) and MD3 (B) of the cross-docked RdCARD-C9CARD complex.

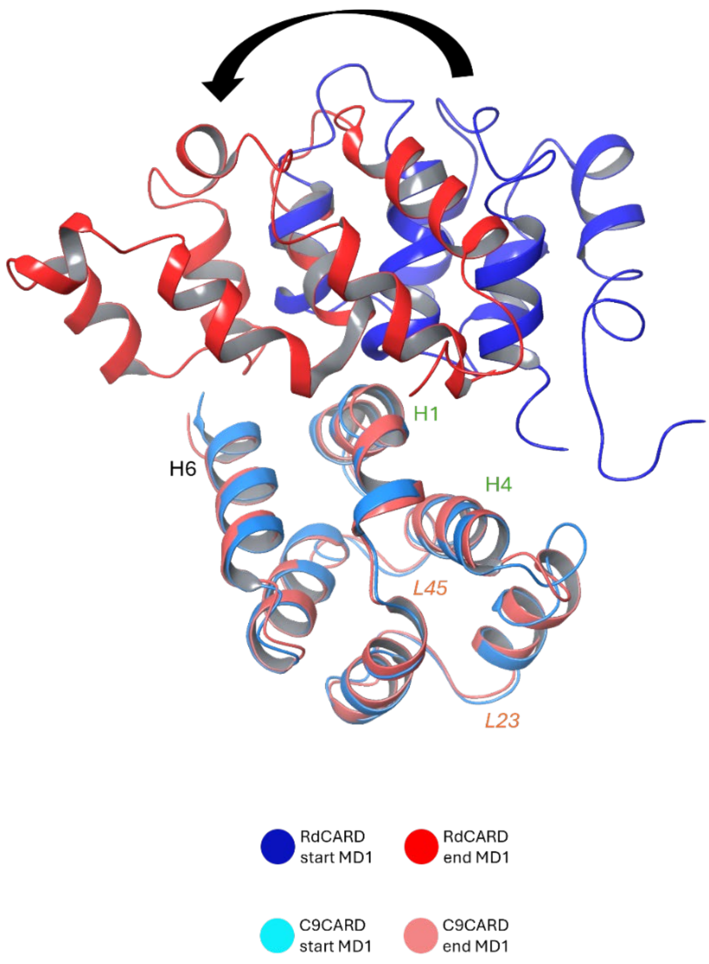

Figure S5. Superposed structure of the RdCARD-C9CARD complex at the start and end of the MD1 simulation, illustrating the displacement of RdCARD away from any known C9CARD binding interface. The green highlights represent the Type I interface helices of the ApCARD-C9CARD complex, while the orange regions indicate the helices and loops involved in the Type II interface of ApCARD-C9CARD. In black, H6 helix, towards which RdCARD shifts during the MD simulation.

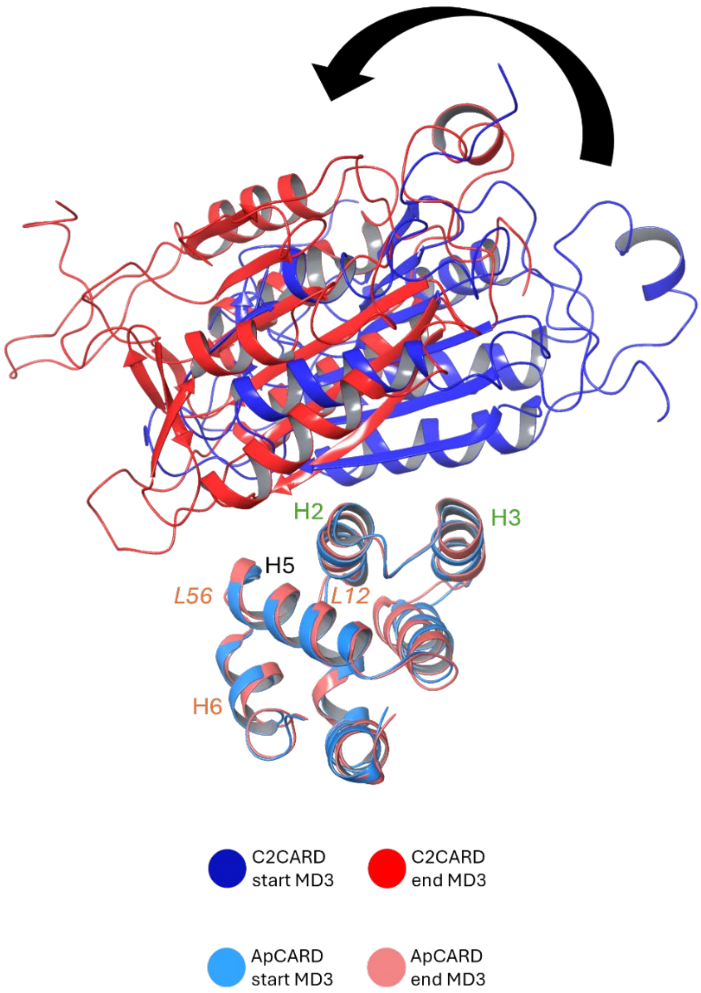

Figure S6. Superposed structure of the ApCARD-C2CARD Cluster 5 complex at the start and end of the MD3 simulation, illustrating the displacement of C2CARD away from any known ApCARD binding interface. The ApCARD helices involved in the Type I interface with C9CARD are shown in green, while the helices and loops engaged in the Type II interface of the ApCARD-C9CARD complex are depicted in orange. In black, H5, towards which C2CARD has shifted during this MD simulation.

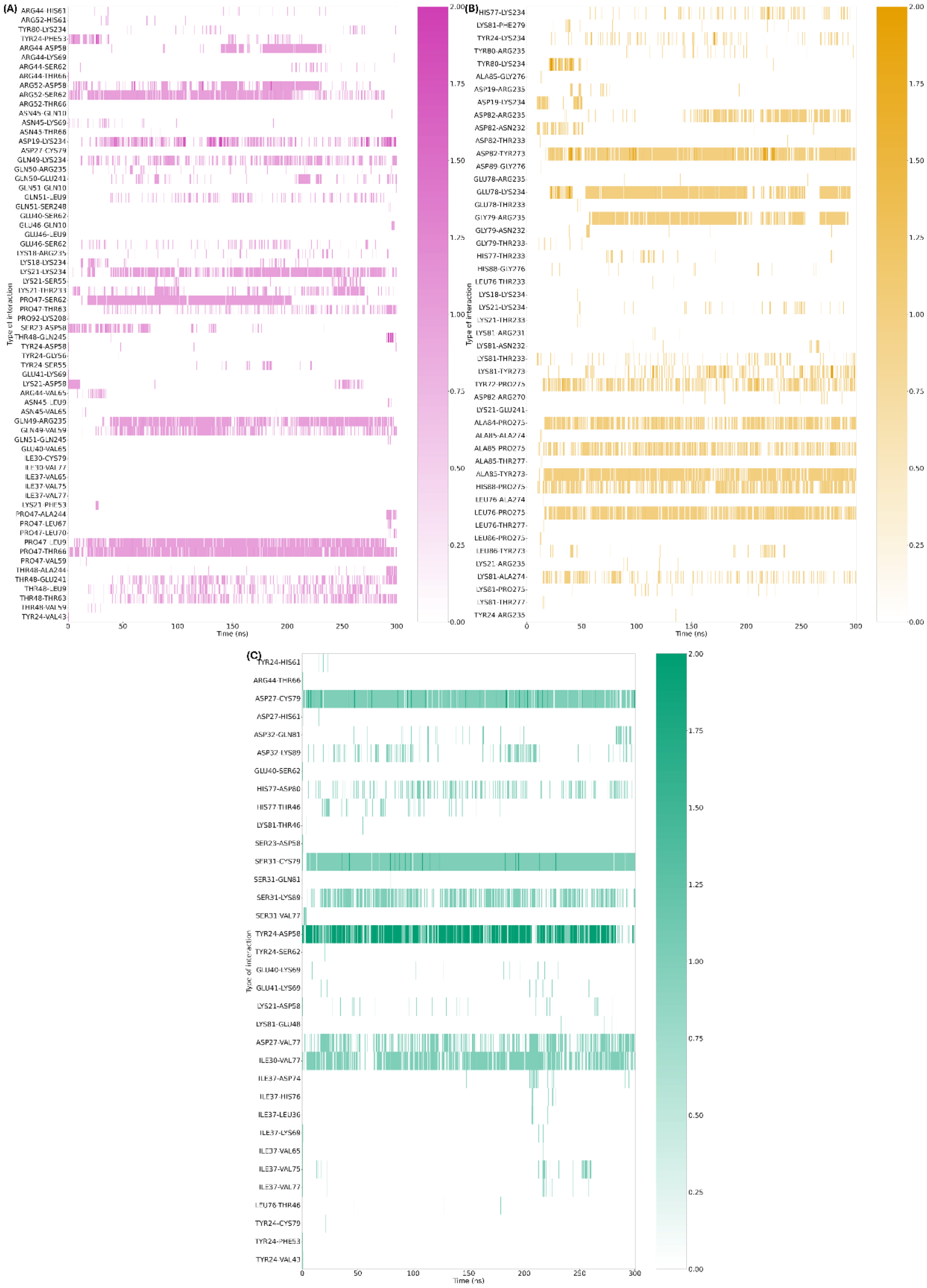

Figure S7. Heatmaps showing the number of interactions by pair of residues as a function of time for the MD simulations of the cross-docked ApCARD-C2CARD Cluster 5 complex: MD1(A), MD2 (B) and MD3 (C).

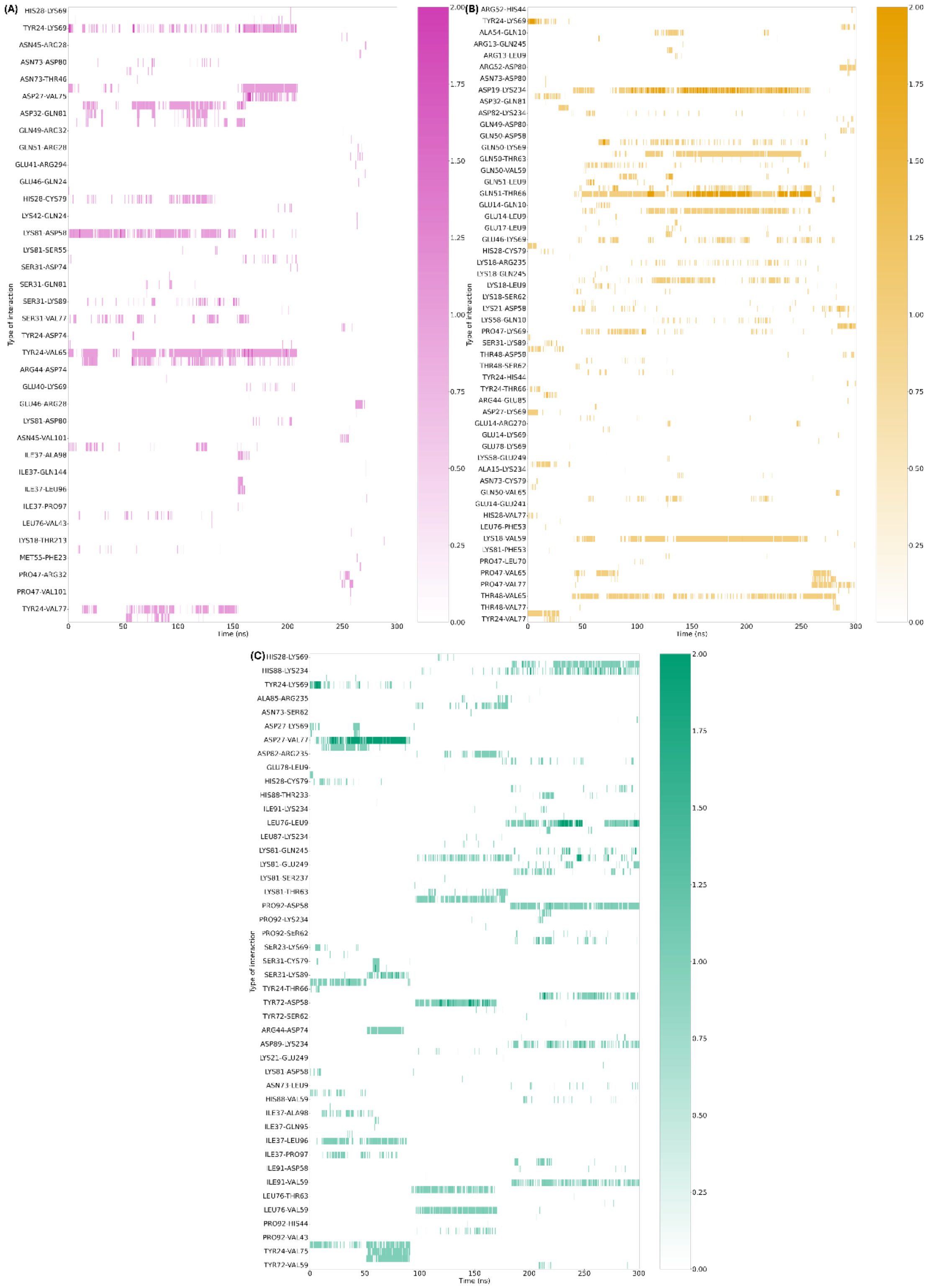

Figure S8. Heatmaps showing the number of interactions by pair of residues as a function of time for the MD simulations of the cross-docked ApCARD-C2CARD Cluster 8 complex: MD1(A), MD2 (B) and MD3 (C).

Seq->	**C9CARD**	*RdCARD*	**ApCARD**	*C2CARD*
**C9CARD**	ID	0,247	0,168	0,06
RdCARD	0,247	ID	0,137	0,138
**ApCARD**	0,168	0,137	ID	0,062
C2CARD	0,06	0,138	0,062	ID

Table S7. Sequence identities of the four CARD domains (ApCARD, C9CARD, C2CARD and RdCARD) calculated based on multiple sequence alignment. The values represent the percentage of identical amino acid residues between each pair of CARD domains.

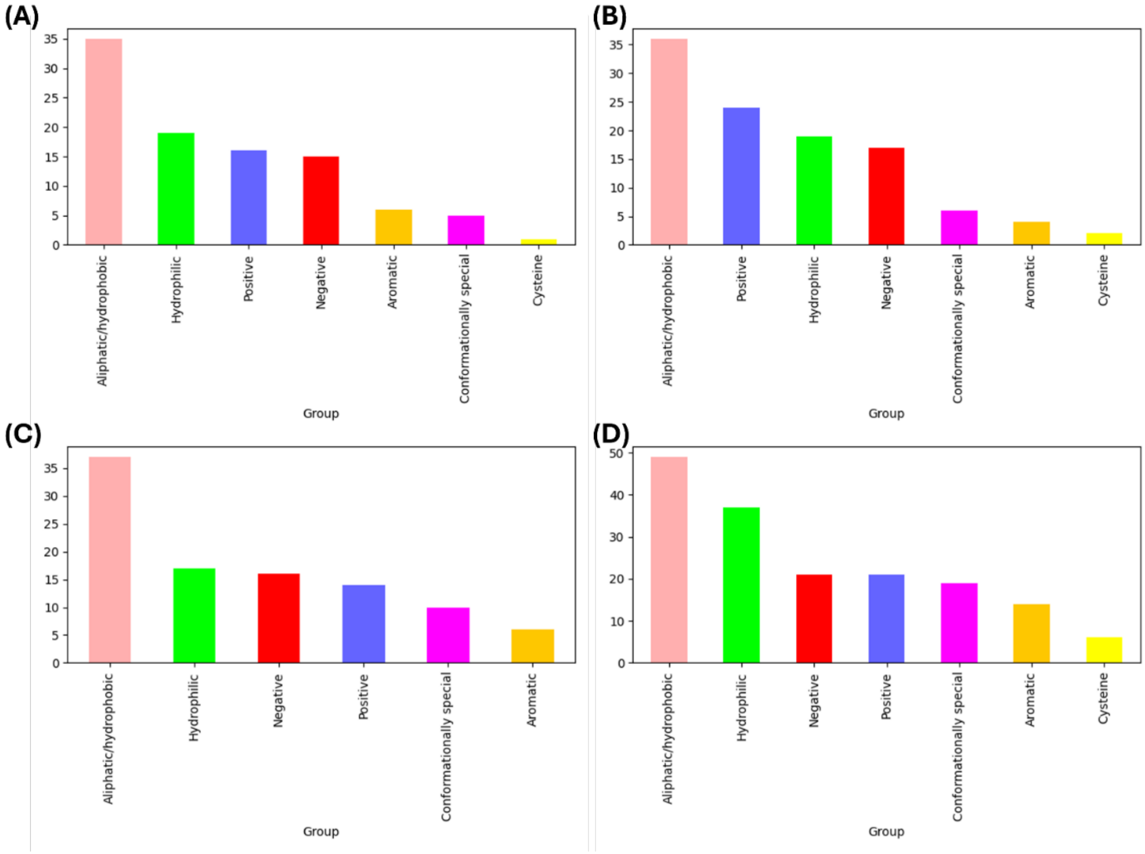

Figure S9. Counts of aminoacids by types following the Zappo classification: (A) ApCARD, (B) C9CARD, (C) RdCARD, (D) C2CARD

References

[1] H. Qin, S. M. Srinivasula, G. Wu, T. Fernandes-Alnemri, E. S. Alnemri, and Y. Shi, "Structural Basis of Procaspase-9 Recruitment by the Apoptotic Protease- Activating Factor 1," Nature, vol. 399, no. 6736, pp. 549–557, 1999, doi: 10.1038/21124.

[2] S. Yuan, M. Topf, T. F. Reubold, S. Eschenburg, and C. W. Akey, "Changes in APAF-1 Conformation that drive Apoptosome Assembly," Biochemistry, vol. 52, no. 13, pp. 2319–2327, 2013, doi: 10.1021/bi301721g.

[3] Q. Hu et al., "Molecular Determinants of Caspase-9 Activation by the APAF-1 Apoptosome," Proceedings of the National Academy of Sciences, vol. 111, no. 46, pp. 16254–16261, Nov. 2014, doi: 10.1073/pnas.1418000111.

[4] T. C. Cheng, C. Hong, I. V Akey, S. Yuan, and C. W. Akey, "A near Atomic Structure of the Active Human Apoptosome," eLife, vol. 5, Oct. 2016, doi: 10.7554/eLife.17755.

[5] T.-W. Su et al., "Structural Insights into DD-Fold Assembly and Caspase-9 Activation by the Apaf-1 Apoptosome," Structure, vol. 25, no. 3, pp. 407–420, Mar. 2017, doi: 10.1016/j.str.2016.12.019.

[6] Y. Li et al., "Mechanistic Insights into Caspase-9 Activation by the Structure of the Apoptosome Holoenzyme," Proceedings of the National Academy of Sciences, vol. 114, no. 7, pp. 1542–1547, Feb. 2017, doi: 10.1073/pnas.1620626114.
